# Identification of MFRP and the secreted serine proteases PRSS56 and ADAMTS19 as part of a molecular network involved in ocular growth regulation

**DOI:** 10.1371/journal.pgen.1009458

**Published:** 2021-03-23

**Authors:** Swanand Koli, Cassandre Labelle-Dumais, Yin Zhao, Seyyedhassan Paylakhi, K. Saidas Nair

**Affiliations:** 1 Department of Ophthalmology, University of California, San Francisco, California, United States of America; 2 Department of Anatomy, University of California, San Francisco, California, United States of America; National Institutes of Health Clinical Center, UNITED STATES

## Abstract

Precise regulation of ocular size is a critical determinant of normal visual acuity. Although it is generally accepted that ocular growth relies on a cascade of signaling events transmitted from the retina to the sclera, the factors and mechanism(s) involved are poorly understood. Recent studies have highlighted the importance of the retinal secreted serine protease PRSS56 and transmembrane glycoprotein MFRP, a factor predominantly expressed in the retinal pigment epithelium (RPE), in ocular size determination. Mutations in *PRSS56 and MFRP* constitute a major cause of nanophthalmos, a condition characterized by severe reduction in ocular axial length/extreme hyperopia. Interestingly, common variants of these genes have been implicated in myopia, a condition associated with ocular elongation. Consistent with these findings, mice with loss of function mutation in PRSS56 or MFRP exhibit a reduction in ocular axial length. However, the molecular network and cellular processes involved in PRSS56- and MFRP-mediated ocular axial growth remain elusive. Here, we show that *Adamts19* expression is significantly upregulated in the retina of mice lacking either *Prss56* or *Mfrp*. Importantly, using genetic mouse models, we demonstrate that while ADAMTS19 is not required for ocular growth during normal development, its inactivation exacerbates ocular axial length reduction in *Prss56* and *Mfrp* mutant mice. These results suggest that the upregulation of retinal *Adamts19* is part of an adaptive molecular response to counteract impaired ocular growth. Using a complementary genetic approach, we show that loss of PRSS56 or MFRP function prevents excessive ocular axial growth in a mouse model of early-onset myopia caused by a null mutation in *Irbp*, thus, demonstrating that PRSS56 and MFRP are also required for pathological ocular elongation. Collectively, our findings provide new insights into the molecular network involved in ocular axial growth and support a role for molecular crosstalk between the retina and RPE involved in refractive development.

## Author summary

During ocular refractive development, the eye’s growth is modulated, such that the ocular axial length matches the optical power enabling the eyes to achieve optimal focus. Alterations in ocular growth mainly contribute to refractive errors. Mutations in human *PRSS56* and *MFRP* are responsible for nanophthalmos that exhibit a severe reduction in ocular axial length, and high hyperopia. Importantly, mutant mouse models lacking either Müller glia expressed PRSS56, or retinal pigment epithelium (RPE) localized MFRP exhibit ocular axial length reduction. Here, we have identified Adamts19 as a factor whose levels were significantly upregulated in the retina of mice lacking either *Prss56* or *Mfrp*. Importantly, utilizing *Adamts19* knockout mice we demonstrate that upregulation of retinal *Adamts19* expression constitutes a compensatory mechanism that provides partial protection against ocular axial reduction due to mutation in *Prss56* and *Mfrp*. Next, utilizing a mouse model of early-onset myopia, we demonstrate that the mutant Irbp induced ocular axial elongation is completely dependent on *Prss56* as well as *Mfrp*, suggesting an interplay between Müller glia and RPE in the regulation of ocular axial growth. Collectively, these findings suggest that ocular refractive development relies on complex interactions occurring between genetic factors in the retina and RPE.

## Introduction

Nanopthalmos is a rare developmental disorder characterized by significantly smaller but structurally normal eyes and extreme hyperopia resulting from compromised ocular growth [1]. In addition, nanophthalmic individuals are highly susceptible to developing blinding conditions including secondary angle-closure glaucoma, spontaneous choroidal effusions, cataracts, and retinal detachment [1]. Both sporadic and familial forms of nanophthalmos with autosomal dominant or recessive inheritance have been described [2]. To date, six genes (*PRSS56*, *MFRP*, *TMEM98*, *CRB1*, *BEST1*, and *MYRF*) have been implicated in familial forms of nanophthalmos, with *PRSS56* and *MFRP* mutations being the most prevalent among multiple cohorts [1,3–10]. Notably, the eyes of nanophthalmic individuals with biallelic mutations in *PRSS56* or *MFRP* were found to be significantly smaller as compared to those carrying dominant mutations in *TMEM98* or *MYRF*. Interestingly, common *PRSS56* and *MFRP* variants have also been found to be associated with myopia, a condition phenotypically opposite to nanophthalmos that is characterized by excessive ocular growth [11,12]. Consistent with human genetic studies, we have demonstrated previously that loss of PRSS56 function in the mouse leads to ocular axial length reduction and hyperopia [13]. Similarly, mice and zebrafish deficient for MFRP exhibit ocular axial length reduction [14–16]. Together, these findings underscore the importance of PRSS56 and MFRP in ocular growth [2].

Ocular growth can be broadly divided into two distinct phases that take place prenatally and postnatally, respectively [17]. Prenatal ocular growth occurs in the absence of visual experience and is primarily dictated by genetic factors [13]. In contrast, postnatal ocular growth also referred to as emmetropization, is a vision-guided process modulated by the refractive status of the eye to ensure that the axial length matches the optical power of the eye to achieve optimal focus and visual acuity. Nanophthalmos is generally attributed to impaired prenatal ocular growth as individuals with this condition are born hyperopic [1,18]. In contrast, common forms of myopia arise from excessive postnatal ocular axial growth. Thus, the association of *PRSS56* and *MFRP* variants with both nanophthalmos and myopia supports a critical role for these factors during distinct phases of ocular growth and suggests that some of the factors and mechanisms regulating ocular growth are conserved between the prenatal and postnatal stages of ocular development.

It is generally accepted that postnatal ocular growth is regulated by a cascade of signaling events by which information is relayed from the retina to the sclera to ultimately induce scleral extracellular matrix (ECM) remodeling and promote ocular axial elongation [19,20]. Notably, *PRSS56* is specifically expressed in the retina [13], which is consistent with a central role for the retina in ocular growth regulation. MFRP is predominantly expressed in the retinal pigment epithelium (RPE) and ciliary epithelium [18]. Importantly, the RPE lies between the retina and the sclera and has been suggested to act as an intermediate in transmitting signals to the sclera during ocular growth [21].

Although current evidence supports a key role for MFRP and PRSS56 in ocular size regulation, the underlying mechanisms remain elusive. In this study, we use *Prss56* and *Mfrp* mutant mouse models and complementary genetic approaches to gain insights into the molecular network involved in ocular size regulation [3,13,22]. Importantly, we identified characteristic changes in the retinal gene expression profile triggered by impaired ocular growth. Specifically, we show that *Adamts19* mRNA levels are significantly increased in the retina of *Prss56* and *Mfrp* mutant mice and provide evidence that the upregulation of retinal *Adamts19* expression is part of an adaptive molecular response to mitigate impaired ocular growth. Moreover, we demonstrate that loss of PRSS56 or MFRP function prevents excessive ocular axial elongation in a mouse model of early-onset myopia caused by a null mutation in *Irbp* [23], further establishing PRSS56 and MFRP as critical regulators of ocular growth. Collectively, our findings identify factors involved in the crosstalk between the retina and the RPE during ocular growth and provide new insight into the molecular network involved in ocular size regulation and refractive development.

## Results

### *Adamts19* expression is upregulated in the retina of *Prss56* mutant mice

**Fig 1:**
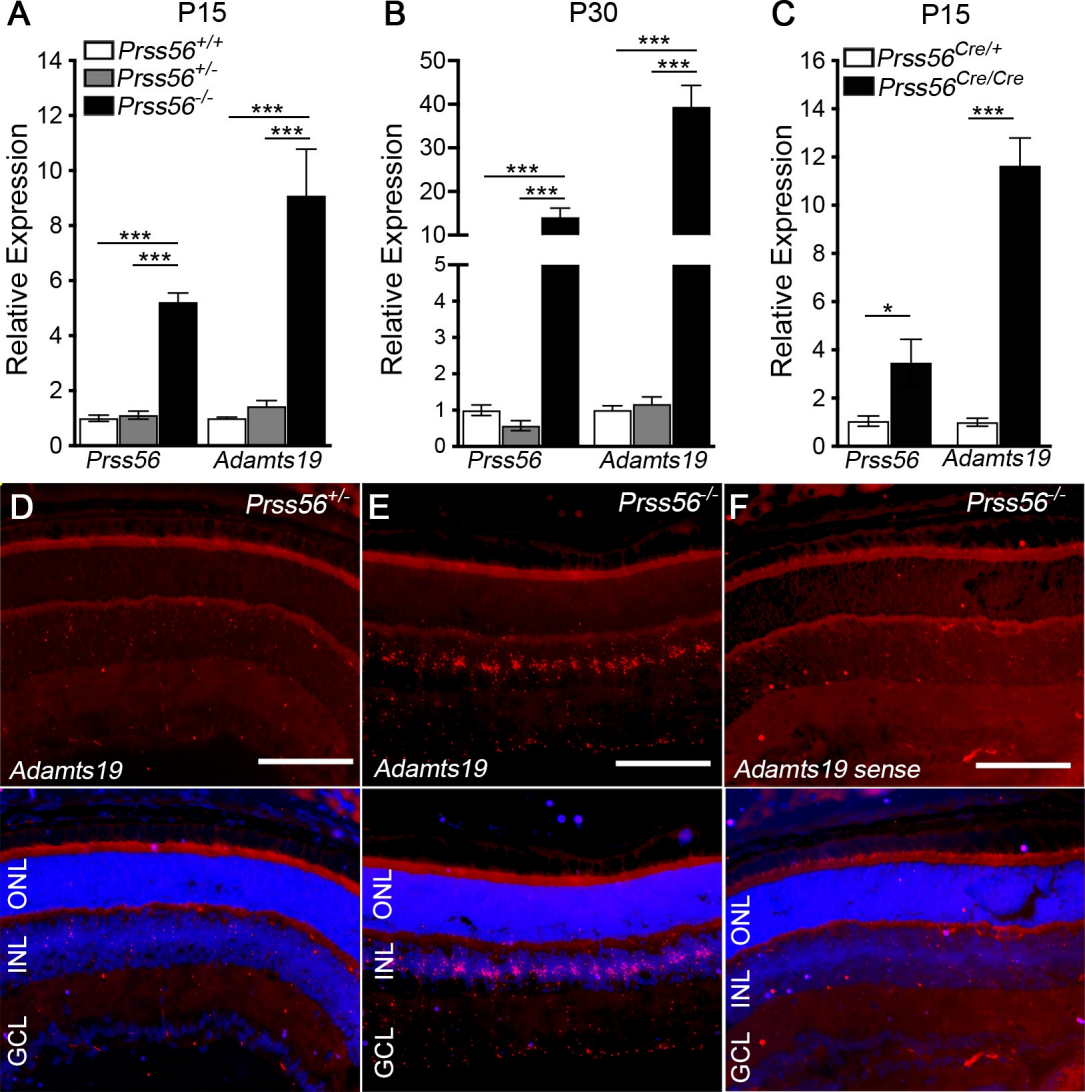
*Adamts19* expression is upregulated in the retina of *Prss56* mutant mice. (**A**-**C**) Graph showing quantification of *Prss56* and *Adamts19* mRNA levels using qPCR in P15 (**A** and **C**) and P30 (**B**) retina from *Prss56*^*glcr4*^ (**A** and **B**) and *Prss56*^*Cre*^ (**C**) mutant strains. While no difference was observed between *Prss56*^*+/+*^ and *Prss56*^*+/glcr4*^ (*Prss56*^*+/-*^) retina, a significant increase in *Prss56* and *Adamts19* mRNA levels was detected in *Prss56*^*glcr4/glcr4*^ (*Prss56*^*-/-*^) retina compared to *Prss56*^*+/+*^ and *Prss56*^*+/glcr4*^ retina at both ages examined (**A** and **B**). Similarly, significant increases in *Prss56* and *Adamts19* mRNA levels were detected in *Prss56*^*Cre/Cre*^ retina compared to the control *Prss56*^*Cre/+*^ retina. *Prss56* and *Adamts19* expression were normalized to the expression of three housekeeping genes (*Hprt1*, *Actb1*, and *Mapk1)*. Data are presented as fold expression relative to wild-type (mean ± SEM), N = 4 to 6 retinas/group, *p<0.05; ***p<0.001, one-way ANOVA (**A** and **B**) or t-test (**C**). (**D-F**) QuantiGene View RNA *in situ* hybridization revealed that *Adamts19* expression was below the threshold level of detection in control *Prss56*^*+/glcr4*^ retina (**D**) and was only detectable in *Prss56*^*glcr4/glcr4*^ retina (**E**) at P16 and no signal was detected with the sense probe (**F**), ocular sections were counterstained with DAPI to visualize nuclei (blue). *Adamts19* expression (red) was predominantly observed in the INL of *Prss56* mutant retina with low levels also detected in the GCL (**E**). Scale bars: 100μm.

To characterize the molecular processes underlying PRSS56-mediated ocular size regulation, we performed a high throughput gene expression analysis on the retina from *Prss56*^*glcr4*^ mutant mice and their control littermates. We demonstrated recently that the *Prss56*^*glcr4*^ mutation, which leads to a truncated PRSS56 protein, caused ocular size reduction via a loss of function mechanism, hence *Prss56*^*glcr4/glcr4*^ mice will be referred to as *Prss56*^*-/-*^ throughout the manuscript [13]. Our transcriptome analysis (RNA-Seq) identified 396 differentially expressed genes, of which 196 were upregulated and 200 downregulated in *Prss56*^*-/-*^ retina (S1 Fig). Fold change analysis revealed that *Prss56* and *Adamts19* were the top two differentially expressed genes between *Prss56* mutant (*Prss56*^-/-^) and control (*Prss56*^+/-^) retina (S1 Table and S1 Data). In addition to being the most significantly upregulated genes, *Prss56* and *Adamts19* both encode secreted serine proteases and might impact similar molecular pathways. Therefore, we focused on these two genes to begin elucidating the mechanisms implicated in ocular size regulation and related diseases. Consistent with the RNA-Seq results, qPCR analysis revealed that *Prss56* and *Adamts19* mRNA levels were significantly upregulated in the retina of *Prss56*^*-/-*^ mice compared to their *Prss56*^*+/-*^ *and Prss56*^*+/+*^ littermates at both ages examined (postnatal day (P) 15 (p = 0.002) and P30 (p = 0.0003) (Fig 1A and 1B). Notably, *Prss56* and *Adamts19* retinal expression levels in *Prss56*^*+/-*^ mice were comparable to those detected in *Prss56*^*+/+*^ mice, which is consistent with the absence of an ocular phenotype in *Prss56*^*+/-*^ mice [13]. *Prss56*^*+/-*^ mice were therefore used as controls for all experiments presented in this study. As described previously, we detected a progressive upregulation of *Prss56* mRNA levels in *Prss56*^*-/-*^ retina from P15 to P60 (S2A Fig) [13]. The increase in *Adamts19* retinal expression was found to precede that of *Prss56* in *Prss56*^*-/-*^ retina, and was detected as early as P10 and gradually increased to reach a peak by P30 (S2B Fig). Upregulation of retinal *Prss56* and *Adamts19* expression was also observed in an alternate genetic mouse model carrying a null allele of *Prss56* (*Prss56*^*Cre*^) we described previously [13], further confirming that the increase in retinal *Prss56* and *Adamts19* expression results from loss of PRSS56 function (Fig 1C). To determine the spatial expression pattern of *Adamts19*, we next performed *in situ* hybridization on ocular sections from *Prss56*^*-/-*^ and *Prss56*^*+/-*^ mice. Despite using the highly sensitive QuantiGene ViewRNA assay, *Adamts19* expression was only detected in *Prss56*^*-/-*^ retina, indicating that *Adamts19* expression was below the threshold of detection in control *Prss56*^*+/-*^ retina (Fig 1D). In *Prss56* mutant retina, *Adamts19* expression was predominantly observed in the inner nuclear layer (INL), a region where the cell bodies of Müller glia are located and which coincides with the retinal expression pattern of *Prss56* (Fig 1E). Collectively, these results demonstrate that in addition to causing ocular size reduction, loss of PRSS56 function leads to alterations in retinal gene expression characterized by increased *Prss56* and *Adamts19* mRNA levels.

### Retinal *Prss56* and *Adamts19* mRNA levels are upregulated in response to ocular size reduction in Prss56 mutant mice

**Fig 2:**
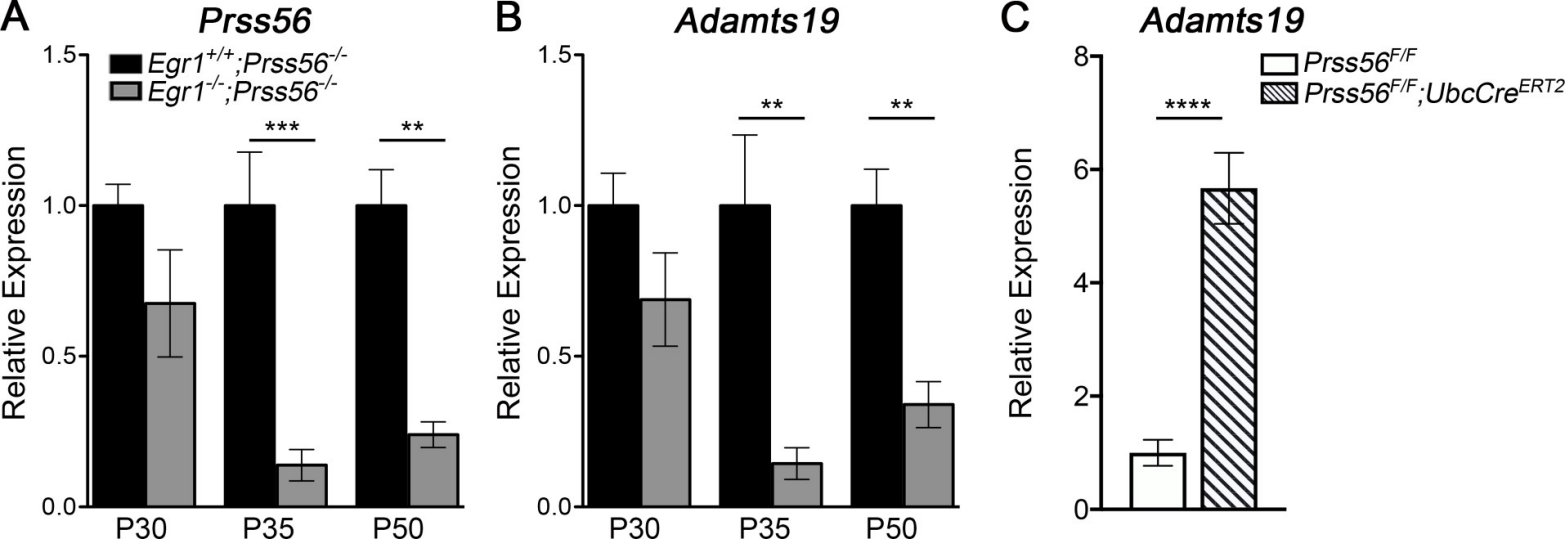
*Egr1* inactivation prevents upregulation of retinal *Prss56* and *Adamts19* expression in *Prss56* mutant mice. (**A**-**B**) Graphs showing quantification of *Prss56* (**A**) and *Adamts19* (**B**) mRNA levels using qPCR in *Prss56* mutant (*Egr1*^*+/+*^*;Prss56*^*-/-*^*)* and *Prss56;Egr1* double mutant (*Egr1*^*-/-*^*;Prss56*^*-/-*^*)* retina at different developmental stages. *Egr1* inactivation reduced retinal *Prss56* (**A**) and *Adamts19* (**B**) mRNA levels in *Prss56* mutant mice (compare *Egr1*^*+/+*^*;Prss56*^*-/-*^ *to Egr1*^*-/-*^*;Prss56*^*-/-*^). (**C**) Graphs showing quantification of *Adamts19* mRNA levels using qPCR analysis in conditional *Prss56* mutant mice in the absence or presence of the inducible ubiquitous *Ubc-Cre* transgene (*Prss56*^*F/F*^ *or Prss56*^*F/F*^;*Ubc-Cre*^*ERT2*^*)* showing upregulation of retinal *Adamts19* following conditional *Prss56* ablation by tamoxifen administration at P21. Data are presented as fold expression relative to wild-type (mean ± SEM), N = 4 to 6 retinas/group. **p<0.01; ***p<0.001, ****p<0.0005 t-test.

To determine whether the upregulation of retinal *Adamts19* and *Prss56* mRNA levels correlates with ocular size reduction in *Prss56* mutant mice, we took advantage of the *Egr1;Prss56* double mutant mouse model (*Egr1*^*-/-*^;*Prss56*^*-/-*^) we described previously [13]. EGR1 (Early Growth Response-1) is a major regulator of ocular growth and *Egr1*^*-/-*^ mice exhibit increased ocular axial length [13,24]. We have previously shown that *Egr1* inactivation rescues the reduction in ocular axial length and vitreous chamber depth (VCD) in *Prss56* mutant mice as the ocular size of *Egr1*^*-/-*^*;Prss56*^*-/-*^ mice is comparable to that of control *Egr1*^*+/-*^*;Prss56*^*+/-*^ mice[13]. Using qPCR analysis, we show that in addition to rescuing ocular axial length reduction, *Egr1* inactivation also prevented the increase in retinal *Prss56 and Adamts19* expression in *Prss56* mutant mice (compare *Egr1*^*-/-*^*;Prss56*^*-/-*^ to *Egr1*^*+/+*^;*Prss56*^*-/-*^ in Fig 2). Notably, *Prss56* and *Adamts19* mRNA levels in *Egr1*^*-/-*^*;Prss56*^*-/-*^ retina were comparable to those observed in control *Egr1*^*+/+*^;*Prss56*^*+/-*^ retina (S3 Fig). These findings suggest that the upregulation of retinal *Prss56* and *Adamts19* expression resulted from the ocular size reduction caused by the loss of PRSS56 function. Using a conditional gene targeting approach, we found that retinal *Adamts19* expression was also increased following conditional inactivation of *Prss56* during the post eye-opening period (P21, Fig 2C). This suggests that *Adamts19* upregulation might play a role at various stages of ocular growth in *Prss56* mutant mice, including the developmental window when the eye is responsive to visual experiences.

### ADAMTS19 is not required for ocular growth during normal development

**Fig 3:**
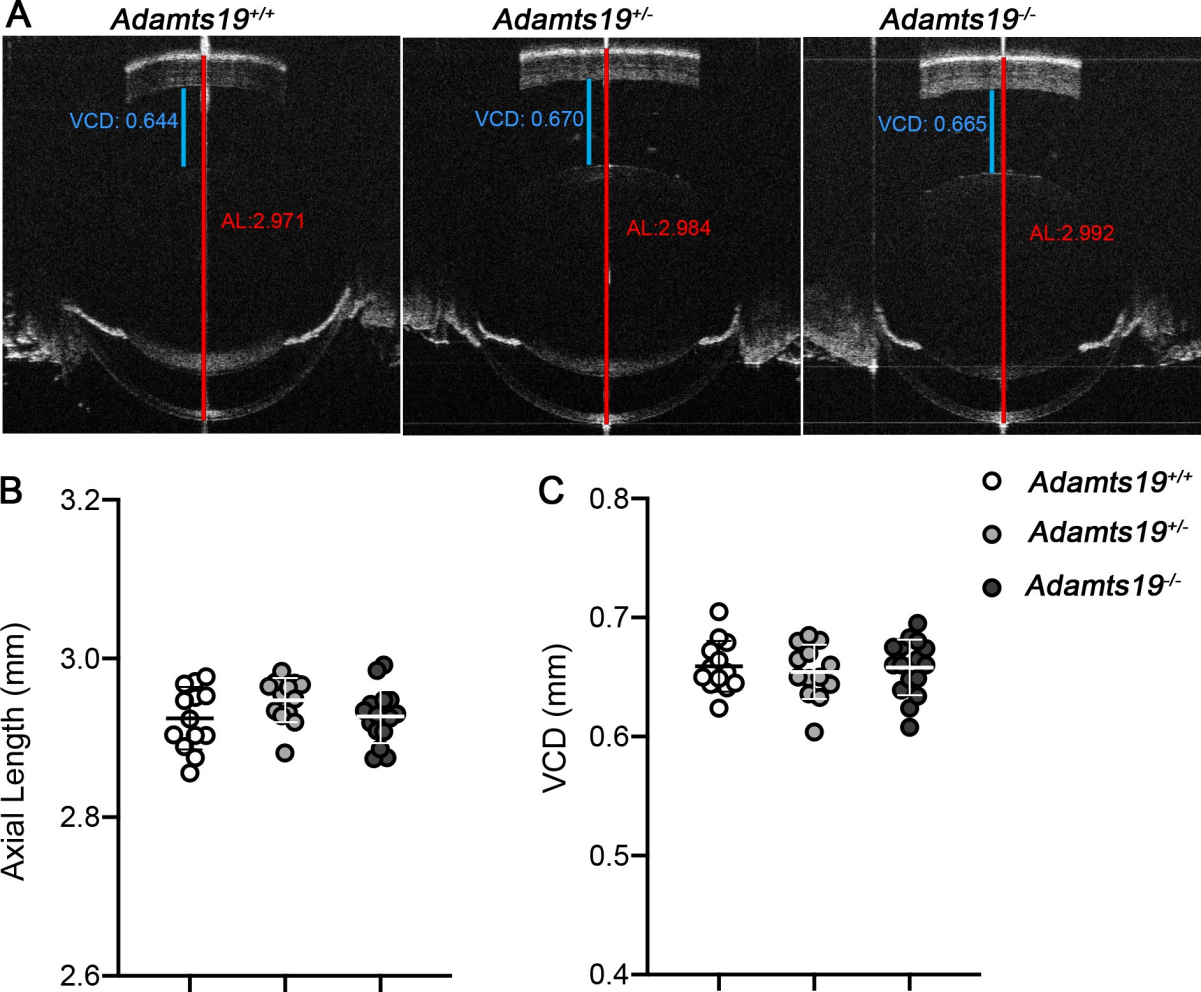
ADAMTS19 is not required for ocular axial growth during normal development. (**A**) Representative OCT images showing that ocular axial length (quantified in **B**) and VCD (quantified in **C**) are indistinguishable between *Adamts19*^*-/-*^, *Adamts19*^*+/-*^ and control *Adamts19*^*+/+*^ mice at P18. Data are presented as mean ± SD, N≥13 eyes/group.

PRSS56 and ADAMTS19 are both secreted serine proteases, raising the possibility that they might have overlapping functions during ocular growth. To test this possibility, we first generated *Adamts19* knockout mice by crossing the conditional *Adamts19* mice with the ubiquitous *β-actin-Cre* line (S4 and S5 Figs). To determine if the loss of ADAMTS19 function leads to ocular defects, we performed spectral-domain optical coherence tomography (SD-OCT) to assess various ocular biometric parameters. We found that all the ocular parameters examined, including ocular axial length, VCD, lens diameter, and retinal thickness were indistinguishable between *Adamts19*^*+/+*^, *Adamts19*^*+/-*^ and *Adamts19*^*-/-*^ mice (Figs 3 and S6). These results demonstrate that ADAMTS19 is not required for ocular growth during normal development.

### *Adamts19* inactivation exacerbates the reduction in ocular axial length in Prss56^-/-^ mice

**Fig 4:**
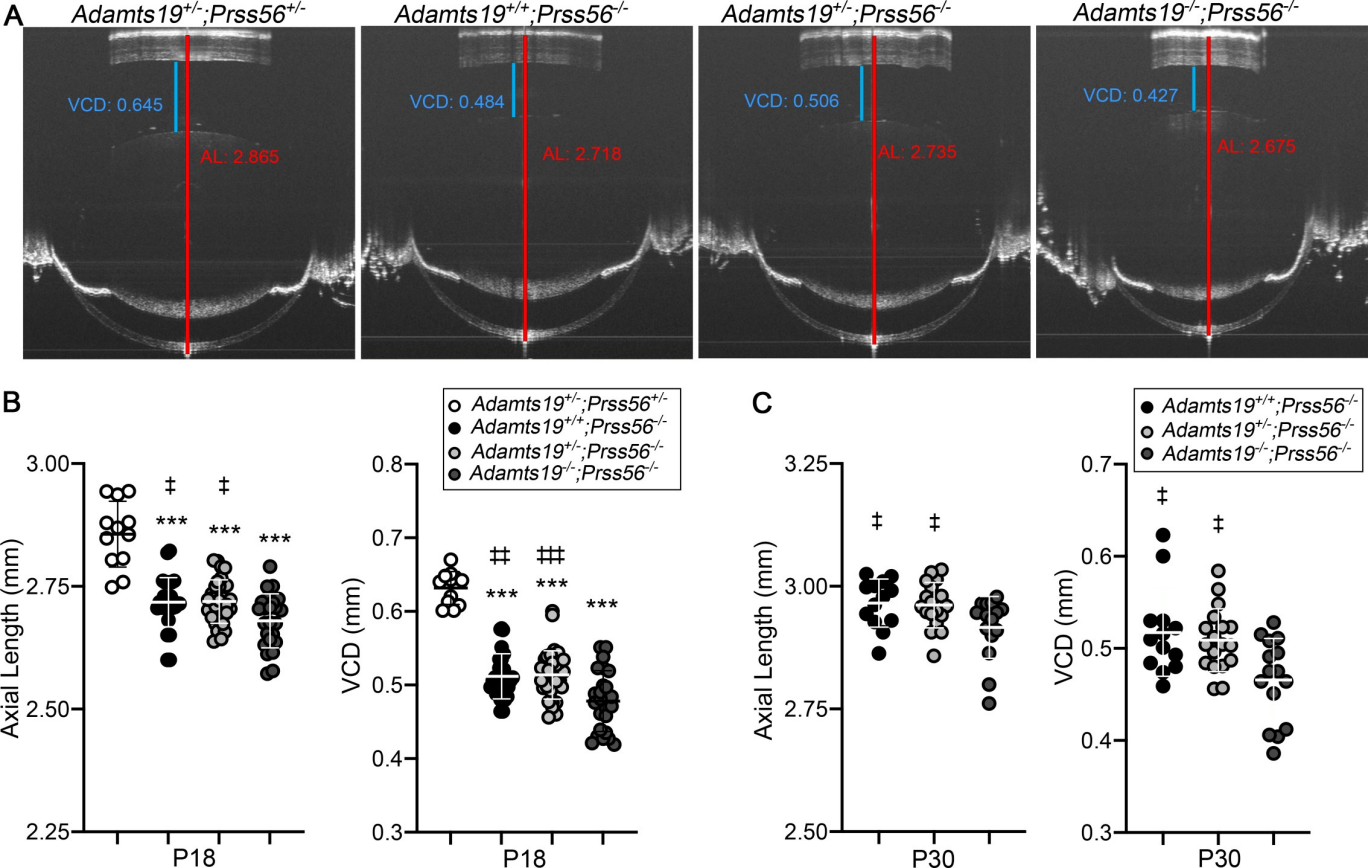
*Adamts19* inactivation exacerbates the reduction in ocular axial length in *Prss56* mutant mice. (**A**) Representative OCT images showing reduced ocular axial length and VCD (quantified in **B** and **C**, respectively) in mice lacking *Prss56* (*Adamts19*^*+/+*^*;Prss56*^*-/-*^, *Adamts19*^*+/-*^*;Prss56*^*-/-*^ and *Adamts19*^*-/-*^*;Prss56*^*-/-*^*)* compared to control *Adamts19*^*+/-*^*;Prss56*^*+/-*^ mice. Importantly, *Adamts19;Prss56* double mutant mice (*Adamts19*^*-/-*^*;Prss56*^*-/-*^) show a modest but consistent reduction in ocular axial length and VCD compared to *Prss56* single mutant mice (*Adamts19*^*+/-*^*;Prss56*^*-/-*^ *or Adamts19*^*+/+*^*;Prss56*^*-/-*^) at both ages examined (P18 in **B** and P30 in **C**). Data are presented as mean ± SD, N≥12 eyes/group. ***p<0.001 (for comparison to *Adamts19*^*+/-*^*;Prss56*^*+/-*^ controls); ^**‡**^p<0.05, ^**‡‡**^p<0.01, ^**‡‡‡**^p<0.001 (for comparison to *Adamts19*^*-/-*^*;Prss56*^*-/-*^ double mutant), one-way ANOVA.

In light of our findings, we hypothesized that the upregulation of retinal *Adamts19* expression might be part of an adaptive molecular response to compensate for the loss of PRSS56 function and promote ocular axial growth. To examine this possibility, we tested the effect of *Adamts19* inactivation in *Prss56*^*-/-*^ mice by crossing *Prss56* mutant mice to the *Adamts19* mutant line to generate *Prss56*^*-/-*^ mice that are wild-type, heterozygous, or homozygous for the *Adamts19* null allele (*Adamts19*^*+/+*^, *Adam19*^*+/-*^ or *Adamts19*^*-/-*^). Since all ocular biometric parameters of *Adamts19*^+/-^; *Prss56*^+/-^ mice were comparable to those of wild-type *(Adamts19*^+/+^;*Prss56*^+/+^) littermates (S7 Fig), they were used as controls. As expected, ocular axial length and VCD were significantly reduced in all three groups of mice lacking *Prss56* (*Prss56*^*-/-*^) compared to the *Adamts19*^+/-^;*Prss56*^+/-^ control mice (Fig 4A and 4B). However, while the lens diameter was comparable between control mice and *Prss56* mutant mice, irrespective of their *Adamts19* genotype (S8A Fig), ocular axial length and VCD were significantly smaller in *Adamts19;Prss56* double mutant mice (*Adamts19*^-/-^;*Prss56*^-/-^) compared to *Adamts19*^*+/-*^;*Prss56*^*-/-*^ or *Adamts19*^*+/+*^;*Prss56*^*-/-*^ at both age examined (p<0.05 and p<0.01 for axial length and VCD at P18 and p<0.05 for axial length and VCD at P30) (Fig 4B and 4C). As reported previously [13], ocular axial length reduction in *Prss56*^*-/-*^ mice was associated with an increase in retinal thickness compared to the *Adamts19*^+/-^;*Prss56*^+/-^ control mice (S8B Fig). Notably, the retina from *Adamts19*^-/-^;*Prss56*^-/-^ mice were modestly but significantly thicker than those from *Adamts19*^*+/+*^; *Prss56*^*-/-*^ mice at P18 (p = 0.0057). In addition, we found that *Adamts19* expression was significantly increased in the retina of both *Adamts19*^*+/+*^;*Prss56*^*-/-*^ and *Adamts19*^*+/-*^;*Prss56*^*-/-*^ mice compared to those of control *Adamts19*^+/-^;*Prss56*^+/-^ mice (p<0.0001 and p = 0.0014, respectively), which is in agreement with the observation that *Adamts19* can partially rescue the reduction in ocular size in *Prss56* mutant mice (S8C Fig). Together, these results demonstrate that *Adamts19* inactivation exacerbates ocular size reduction in *Prss56*^*-/-*^ mice and is consistent with the upregulation of retinal *Adamts19* expression being part of an adaptive molecular response triggered by impaired ocular growth in *Prss56* mutant mice.

### *Adamts19* inactivation exacerbates the reduction in ocular axial length in *Mfrp* mutant mice

**Fig 5:**
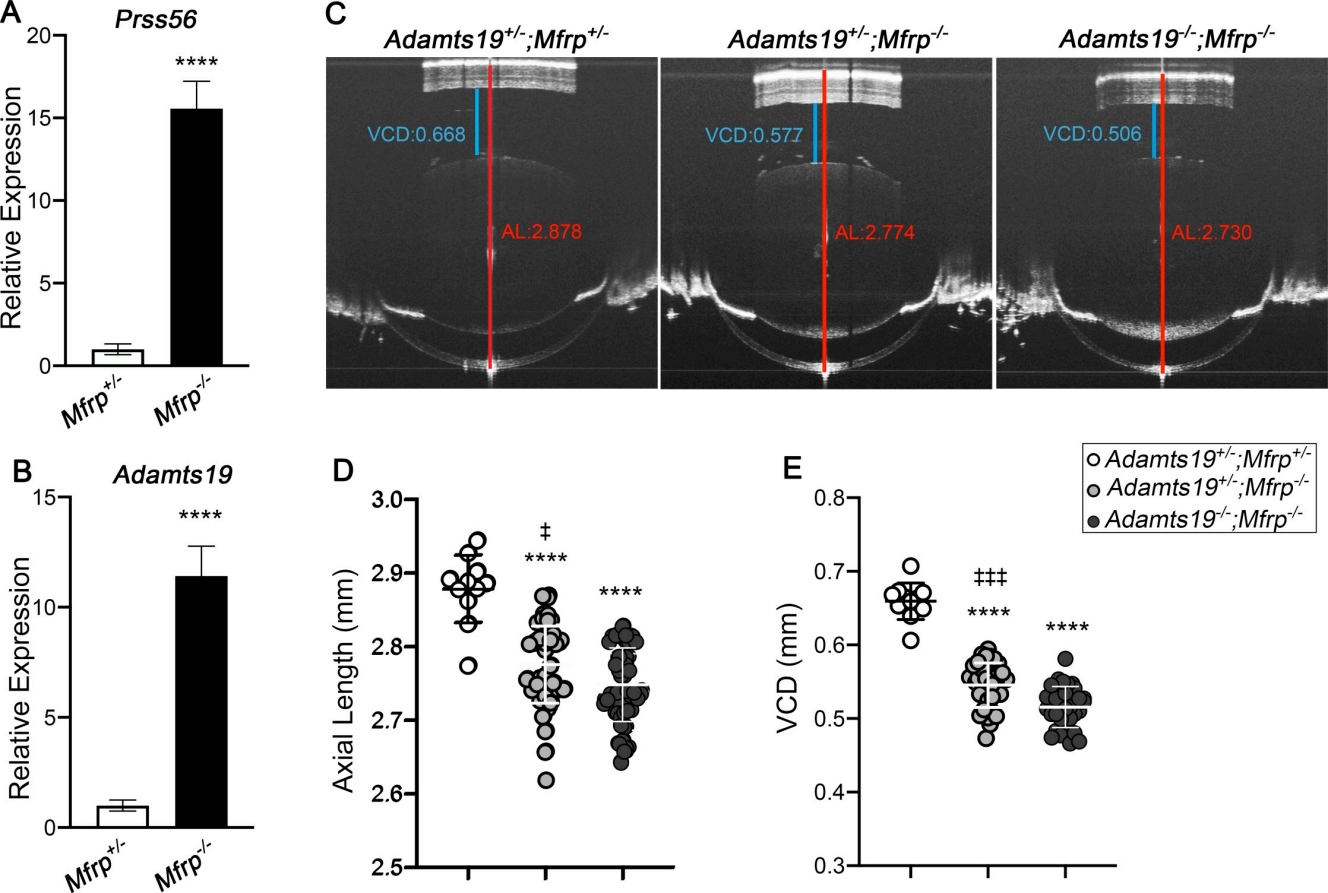
*Adamts19* inactivation exacerbates the reduction in ocular axial length in *Mfrp* mutant mice. (**A**-**B**) Histogram showing relative *Prss56 (***A**) and *Adamts19* (**B**) mRNA levels in *Mfrp*^*+/-*^ and *Mfrp*^*-/-*^ retina at P18. A significant increase in *Prss56 (***A**) and *Adamts19* (**B**) was detected in *Mfrp*^*-/-*^ compared to *Mfrp*^*+/-*^ retina, N≥4 retinas/group. (**C**) Representative OCT images showing that ocular axial length (quantified in **D**) and VCD (quantified in **E**) are reduced in *Mfrp* single mutant *(Adamts19*^*+/-*^;*Mfrp*^*-/-*^) and *Adamts19;Mfrp* double mutant *(Adamts19*^*-/-*^;*Mfrp*^*-/-*^*)* mice compared to control *(Adamts19*^*+/-*^;*Mfrp*^*+/-*^) mice at P18, N≥11 eyes/group. Of note, *Adamts19*^*-/-*^;*Mfrp*^*-/-*^ mice show a modest but significant reduction in axial length and VCD compared to *Adamts19*^*+/-*^;*Mfrp*^*-/-*^ mice. Data are presented as mean ± SEM (**A** and **B**) or mean± SD (**D** and **E**). ****p<0.0001 (for comparison to *Adamts19*^*+/-*^;*Mfrp*^*+/-*^ controls); ^**‡**^p<0.05, ^**‡‡‡**^p<0.001 (for comparison to *Adamts19*^*-/-*^;*Mfrp*^*-/-*^ mutant), one-way ANOVA.

Interestingly, elevated levels of retinal *Prss56* expression have recently been reported in another mouse model of nanophthalmos caused by a mutation in the gene coding for membrane frizzed related-protein (*Mfrp*) [22]. Although increased *Adamts19* expression was also reported in *Mfrp*^*-/-*^ eyes, the specific ocular tissue/cell type expressing *Adamts19* was not addressed [22]. Since *Adamts19* expression was specifically detected in the retina of *Prss56*^*-/-*^ mice, we performed a qPCR analysis to confirm that *Prss56* and *Adamts19* mRNA levels were upregulated in the retina from *Mfrp*^*-/-*^ mice compared to control *Mfrp*^*+/-*^ littermates (Fig 5A). To determine if *Adamts19* inactivation also exacerbates the ocular size reduction caused by *Mfrp* deficiency, we crossed *Mfrp* mutant mice with the *Adamts19* mutant line and conducted SD-OCT analyses on the progeny. Since the ocular biometric parameters of *Adamts19*^*+/-*^*;Mfrp*^*+/-*^ mice were comparable to those of wild-type mice (*Adamts19*^*+/+*^*;Mfrp*^*+/+*^), they were used as controls (S9 Fig). As expected, ocular axial length and VCD were significantly reduced in *Mfrp* mutant mice (*Adamts19*^*+/-*^*;Mfrp*^*-/-*^) compared to *Adamts19*^*+/-*^*;Mfrp*^*+/-*^ control littermates, and the lens diameter was comparable across the groups (Figs 5C–5E and S10A). Importantly, the ocular axial length and VCD were further reduced in *Adamts19*^*-/-*^*;Mfrp*^*-/-*^ mice compared to *Mfrp* mutant mice (*Adamts19*^*+/-*^*;Mfrp*^*-/-*^*)* (p = 0.0388 and p = 0.0001, respectively) (Fig 5C–5E). Additionally, retinal thickness was significantly increased in *Adamts19*^*+/-*^*;Mfrp*^*-/-*^ and *Adamts19*^*-/-*^*;Mfrp*^*-/-*^ mice compared to control *Adamts19*^*+/-*^*; Mfrp*^*+/-*^ mice (p<0.0001) (S10B Fig). These findings further support a role for the upregulation of retinal *Adamts19* expression being part of a compensatory mechanism triggered by impaired ocular axial growth.

### Inactivation of *Prss56* or *Mfrp* prevents pathological ocular axial elongation in Irbp mutant mice

**Fig 6:**
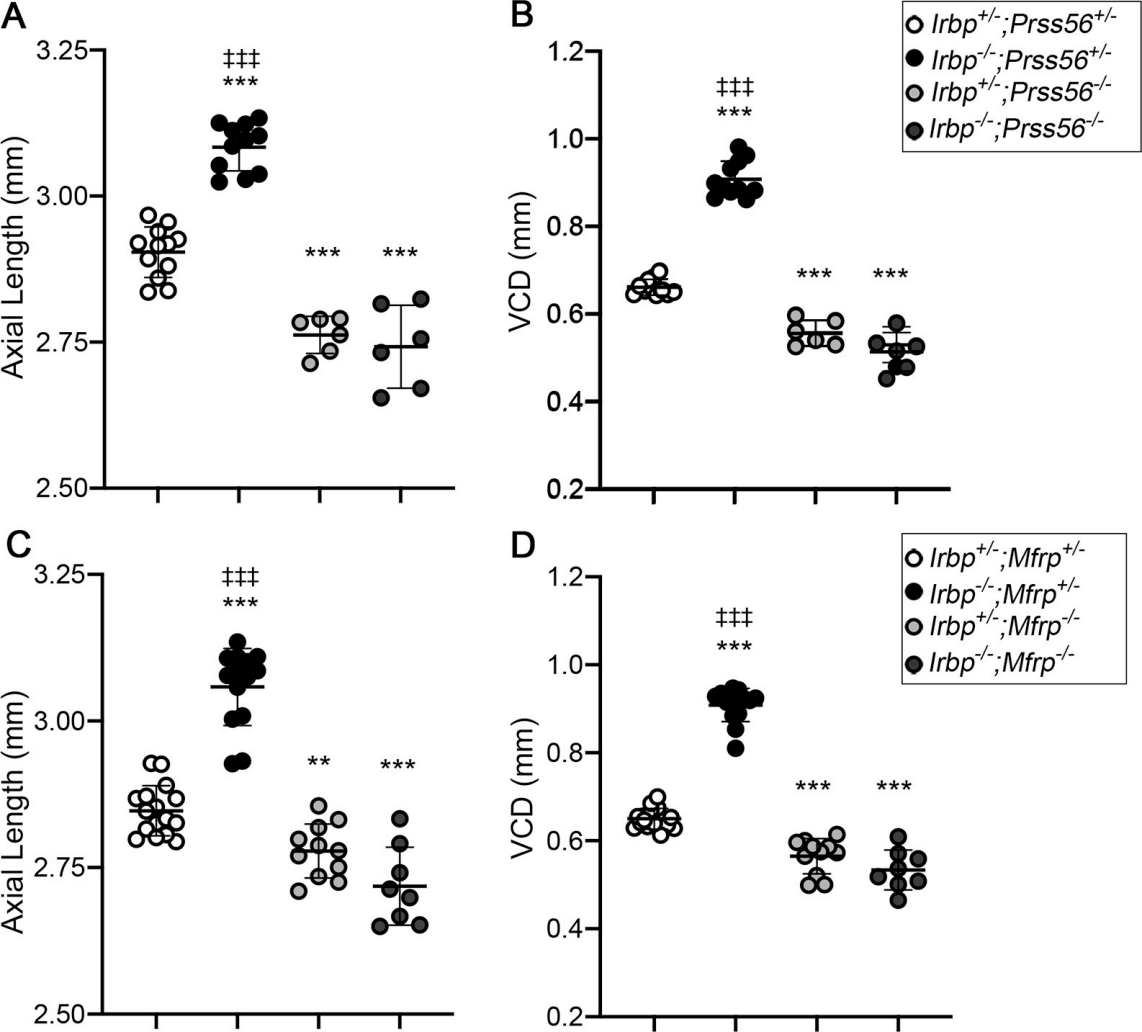
Excessive ocular axial elongation in *Irbp* mutant mice is dependent on PRSS56 and MFRP. (**A**-**D**) Scatter plot showing quantification of ocular axial length (**A** and **C**) and VCD (**B** and **D**) in *Irbp* mutant mice carrying heterozygous or homozygous mutations in *Prss56* or *Mfrp*. Biometric analyses revealed that ocular axial length (**A** and **C**) and VCD (**B** and **D**) were significantly increased in *Irbp* mutant mice (*Irbp*^*-/-*^;*Prss56*^*+/-*^ in **A** and **B,** and *Irbp*^*-/-*^*;Mfrp*^*+/-*^ in **C** and **D**) and significantly reduced in *Prss56* and *Mfrp* single mutant mice (*Irbp*^*+/-*^;*Prss56*^*-/-*^ in **A** and **C** and *Irbp*^*+/-*^; *Mfrp*^*-/-*^ in **B** and **D,** respectively) compared to their respective control littermates (*Irbp*^*+/-*^;*Prss56*^*+/-*^ or *Irbp*^*+/-*^;*Mfrp*^*+/-*^). Notably, *Prss56* and *Mfrp* inactivation prevented ocular axial elongation in *Irbp* mutant mice, as ocular axial length and VCD of *Irbp*^*-/-*^;*Prss56*^*-/-*^ and *Irbp*^*-/-*^; *Mfrp*^*-/-*^ are comparable to those of *Prss56* or *Mfrp* single mutants (*Irbp*^*+/-*^;*Prss56*^*-/-*^ and *Irbp*^*+/-*^; *Mfrp*^*-/-*^, respectively). Data are presented as mean ± SD (**A-D**), N≥6 eyes/group. **p<0.01; ***p<0.001 (for comparison to control *Irbp*^*+/-*^;*Prss56*^*+/-*^ or *Irbp*^*+/-*^;*Mfrp*^*+/-*^ mice); ^**‡‡‡**^p<0.001 (for comparison to *Irbp*^*-/-*^;*Prss56*^*-/-*^ or *Irbp*^*-/-*^; *Mfrp*^*-/-*^ mice), one-way ANOVA.

PRSS56 and MFRP are required for ocular axial growth during normal development [3,6,13,18]. To determine if these factors are also implicated in pathological ocular elongation, we tested the effects of *Prss56* and *Mfrp* inactivation in a mouse model of early-onset myopia associated with excessive ocular axial growth that is caused by a null mutation in the gene coding for IRBP (Interphotoreceptor retinoid-binding protein) [23]. To this end, each of the *Prss56* and *Mfrp* mutant lines were crossed to *Irbp* mutant mice and biometric ocular assessment was conducted on the progeny. Since the ocular biometric parameters of double heterozygous mice (*Irbp*^*+/-*^*;Prss56*^*+/-*^ and *Irbp*^*+/-*^*;Mfrp*^*+/-*^) were comparable to those of wild-type mice (*Irbp*^*+/+*^*;Prss56*^*+/+*^ and *Irbp*^*+/+*^*;Mfrp*^*+/+*^), they were used as controls (S11 Fig). As expected, SD-OCT analyses revealed that both the ocular axial length and VCD were significantly increased in *Irbp* single mutant mice (*Irbp*^*-/-*^*;Prss56*^*+/-*^ or *Irbp*^*-/-*^*;Mfrp*^*+/-*^) (p<0.0001) and significantly reduced in *Prss56 or Mfrp* single mutant mice (*Irbp*^*+/-*^*;Prss56*^*-/-*^ or *Irbp*^*+/-*^*;Mfrp*^*-/-*^) (p<0.001 for axial length and VCD in *Prss56* single mutants and p = 0.01 and p<0.001 for axial length and VCD in *Mfrp* single mutants) compared to their respective controls (*Irbp*^*+/-*^*;Prss56*^*+/-*^ and *Irbp*^*+/-*^*;Mfrp*^*+/-*^ mice) (Figs 6A–6D, S12A and S13A). Inactivation of either *Prss56* or *Mfrp* prevented ocular axial elongation in *Irbp* mutant mice (*Irbp*^*-/-*^*;Prss56*^*-/-*^ and *Irbp*^*-/-*^*;Mfrp*^*-/-*^, respectively). Notably, ocular axial length and VCD were significantly reduced in both double mutant lines (*Irbp*^*-/-*^*;Prss56*^*-/-*^ and *Irbp*^*-/-*^*;Mfrp*^*-/-*^) (p<0.001) compared to their respective control littermates (*Irbp*^*+/-*^*;Prss56*^*+/-*^ and *Irbp*^*+/-*^*;Mfrp*^*+/-*^) and were comparable to those observed in *Prss56* and *Mfrp* single mutant mice (*Irbp*^*+/-*^*;Prss56*^*-/-*^ and *Irbp*^*+/-*^*;Mfrp*^*-/-*^, respectively). In addition, while retinal thickness was increased in both *Prss56* and *Mfrp* single mutant mice (*Irbp*^*+/-*^*;Prss56*^*-/-*^ and *Irbp*^*+/-*^*;Mfrp*^*-/-*^), it was significantly reduced in *Irbp* single mutant mice (*Irbp*^*-/-*^*;Prss56*^*+/-*^ or *Irbp*^*-/-*^*;Mfrp*^*+/-*^) compared to control littermates (*Irbp*^*+/-*^*;Prss56*^*+/-*^ and *Irbp*^*+/-*^*;Mfrp*^*+/-*^, respectively) (p<0.0001) (S12B and S13B Figs). Consistent with the observed reduction in their ocular size, the retinal thickness of *Irbp*^*-/-*^*;Prss56*^*-/-*^ and *Irbp*^*-/-*^*;Mfrp*^*-/-*^ mice was comparable to that of their respective *Prss56* and *Mfrp* single mutant littermates (*Irbp*^*+/-*^*;Prss56*^*-/-*^ and *Irbp*^*+/-*^*;Mfrp*^*-/-*^). Together, these findings demonstrate that the excessive ocular elongation observed in *Irbp*^*-/-*^ mice is dependent on PRSS56 and MFRP functions and suggest that IRPB, PRSS56, and MFRP are likely part of the same pathway contributing to ocular growth.

**Fig 7:**
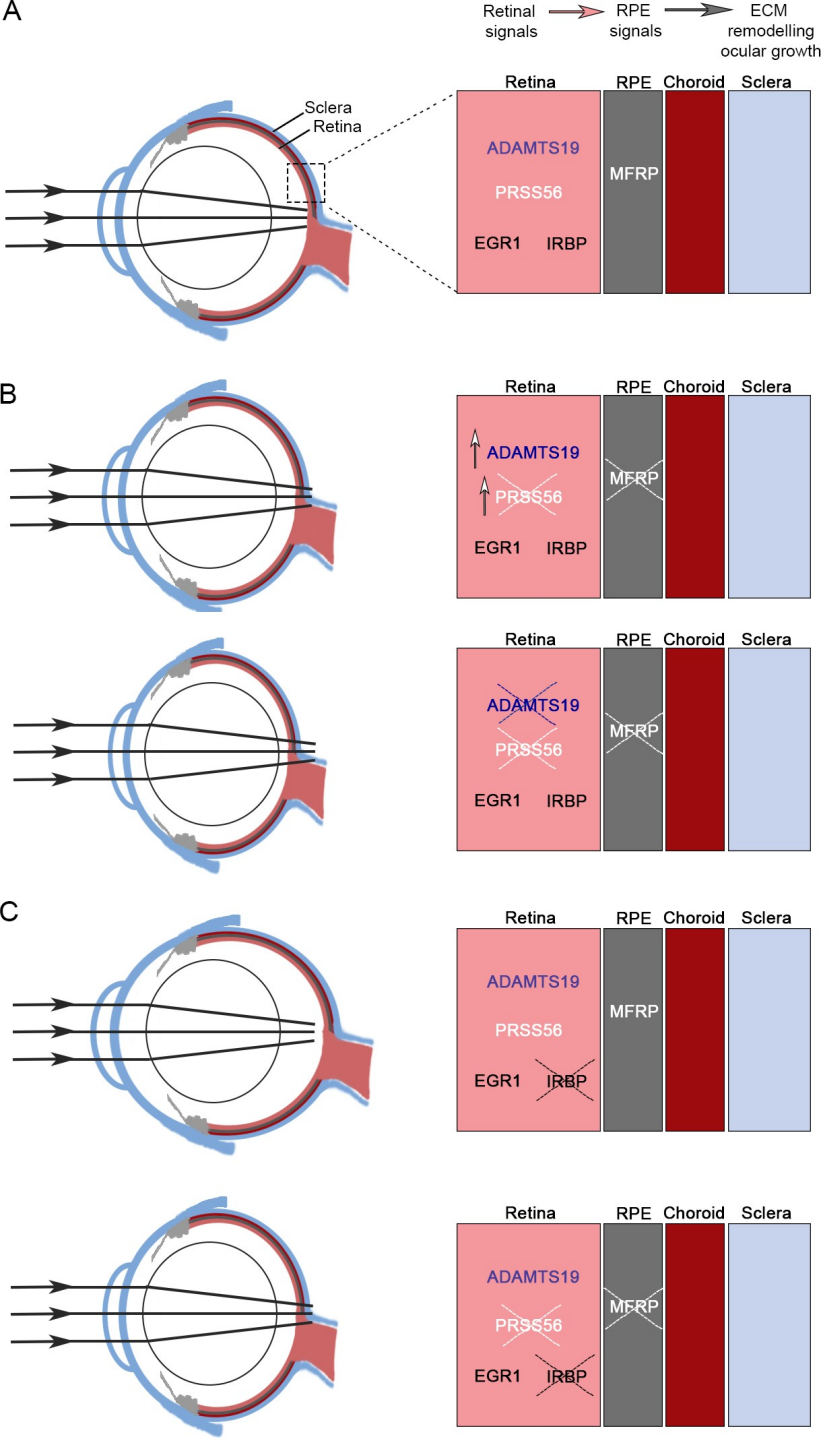
Summary of the molecular network involved in normal and excessive ocular axial growth. (**A-C**) Schematic representations of emmetropic (**A**), hyperopic (**B**), and myopic (**C**) mouse eyes and a summary of corresponding molecular correlates associated with ocular growth described in this study. It is generally accepted that ocular growth relies on a cascade of signaling events originating from the retina that are sequentially transmitted to the sclera to promote scleral ECM remodeling and ocular elongation. During postnatal developmental stages in humans and the post eye-opening period in the mouse, ocular growth is guided by visual cues to ensure that the optical power of the eye matches its axial length, a state referred to as emmetropia (**A**). Genetic inactivation of *Prss56* or *Mfrp* leads to a significant reduction in ocular axial length causing the focused image to fall beyond the retina (hyperopia) (**B**). Retinal *Adamts19* mRNA was significantly upregulated in response to impaired ocular growth caused by mutations in *Prss56* or *Mfrp*. Although ADAMTS19 is not required for ocular growth during normal development, its inactivation exacerbates ocular axial length reduction in *Prss56* and *Mfrp* mutant mice. Additionally, inactivation of *Prss56* or *Mfrp* prevented excessive ocular elongation in a mouse model of early-onset myopia caused by a null mutation in *Irbp*. Notably, *Prss56* and *Mfrp* were found to be epistatic to *Irbp* during ocular elongation as shown by the ocular size of *Irbp;Prss56* and *Irbp;Mfrp* double mutant mice being comparable to that of *Prss56* and *Mfrp* single mutants (**C**). Overall, findings from this study show that PRSS56 and MFRP promote ocular growth during both normal and pathophysiological developmental processes.

To further investigate the relationship between IRBP and PRSS56 during ocular elongation, we next assessed the retinal expression of *Adamts19* in *Irbp* mutant mice carrying various *Prss56* genotypes. Notably, the qPCR analysis revealed that the retinal expression of *Adamts19* in double heterozygous (*Irbp*^*+/-*^*;Prss56*^*+/-*^) mice was comparable to that of wild-type mice (*Irbp*^*+/+*^*;Prss56*^*+/+*^, S14 Fig), which is consistent with the absence of an ocular phenotype in *Irbp*^*+/-*^*;Prss56*^*+/-*^ mice (Figs 6 and S11). As expected, *Adamts19* mRNA levels were significantly upregulated in *Prss56* mutant retina (*Irbp*^*+/-*^*;Prss56*^*-/-*^) compared to *Irbp*^*+/+*^*;Prss56*^*+/+*^ and *Irbp*^*+/-*^*;Prss56*^*+/-*^ control retinas. Interestingly, while no change in retinal *Adamts19* expression was detected in *Irbp* mutant mice carrying two wild-type *Prss56* alleles (*Irbp*^*-/-*^*;Prss56*^*+/+*^), it was upregulated in *Irbp*^*-/-*^*;Prss56*^*+/-*^ mice, although to a lesser extent of what is observed in *Prss56* mutant mice. These results suggest that the upregulation of retinal *Adamts19* expression might be an attempt to compensate for the loss of one functional copy of *Prss56* and might contribute to the excessive ocular elongation in *Irbp*^*-/-*^*;Prss56*^*+/-*^ mice. Furthermore, retinal expression of *Adamts19* was significantly higher in double mutants (*Irbp*^*-/-*^*;Prss56*^*-/-*^) compared to *Irbp* or *Prss56* single mutant mice (*Irbp*^*-/-*^*;Prss56*^*+/-*^ and *Irbp*^*+/-*^*;Prss56*^*-/-*^, respectively) suggesting that *Adamts19* upregulation induced by loss of PRSS56 function is exacerbated by *Irbp* inactivation. Overall, our data suggest that increased *Adamts19* upregulation is achieved through distinct mechanisms to mitigate the effect of molecular defects leading to impaired ocular axial growth (Fig 7A and 7B).

## Discussion

The molecular and cellular mechanisms involved in ocular axial growth and emmetropization are poorly understood. Previous studies have identified *PRSS56* and *MFRP* mutations as a major cause of nanophthalmos, a condition characterized by severe ocular size reduction and extreme hyperopia, suggesting that these factors play a critical role in ocular growth [3–6]. Consistent with this, *Prss56* and *Mfrp* mutant mice recapitulate the cardinal features of nanophthalmos, i.e. reduced ocular axial length and hyperopia [3,13,16]. Here, we use complementary genetic strategies in *Prss56* and *Mfrp* mutant mouse models as a first step to elucidate the molecular and cellular networks implicated in ocular size determination. Notably, we identified ADAMTS19 as a novel factor involved in ocular size regulation and demonstrate that the upregulation of retinal *Adamts19* expression is part of a protective molecular response to impaired ocular growth. Furthermore, we show that inactivation of *Prss56* or *Mfrp* prevent excessive ocular elongation in a mouse model of developmental myopia caused by a null mutation in *Irbp*, demonstrating that PRSS56 and MFRP are not only essential for ocular growth during normal development but also required to induce ocular elongation associated with early-onset/developmental myopia.

Using two distinct mouse models of nanophthalmos and a combination of genetic approaches, we identified a retinal gene expression signature characterized by the upregulation of the genes coding for PRSS56 and ADAMTS19 that is associated with the reduced ocular size. Notably, RNA-Seq analysis revealed that *Prss56* and *Adamts19* were the top two differentially expressed genes between *Prss56* mutant (*Prss56*^-/-^) and control (*Prss56*^+/-^) retina. Besides *Prss56*, *Tmem98* was the only other differentially expressed gene that has previously been implicated in nanophthalmos [8,25] and was found to be significantly downregulated in the retina from *Prss56*^*-/-*^ mice compared to control *Prss56*^*+/-*^ mice (S1 Table). Interestingly, *TMEM98*, which encodes a single transmembrane protein of unknown function, is also one of the several loci associated with high myopia in genome-wide association study (GWAS) [12]. Notably, conditional ablation of *Tmem98* from the RPE, leads to significant increase in ocular size and axial length [8]. Thus, it is possible that the reduced *Tmem98* expression detected in *Prss56*^*-/-*^ mice might have resulted from the differential expression in the fraction of RPE attached to the isolated retina for RNA-Seq analysis. In which case *Tmem98* downregulation could be part of a molecular response resulted to counteract impaired ocular elongation in *Prss56*^*-/-*^ mice.

Importantly, we show that although ADAMTS19 is not required for ocular axial growth during normal development, its inactivation exacerbates the reduction in ocular axial length and VCD in both *Prss56* and *Mfrp* mutant mouse models. Of note, the presence of a single wild-type *Adamts19* allele (*Adamts19*^*+/-*^) is sufficient to confer a protective effect against axial length reduction in *Prss56* and *Mfrp* mutant mice. In addition to ocular axial length reduction, *Mfrp* mutant mice also develop retinal degeneration by 8 to 10 weeks of age [26]. Since ocular axial length reduction is detected before any noticeable retinal morphological changes are observed, it is likely that ocular size reduction and retinal degeneration are independent insults. Thus, these results suggest that the upregulation of retinal *Adamts19* expression constitutes a protective molecular response to overcome impaired ocular axial growth.

Our *in situ* hybridization analysis revealed that *Adamts19* expression was predominantly observed in the retinal INL in *Prss56*^*-/-*^ eyes and was below the threshold of detection in wild-type eyes, which is in agreement with the absence of ocular phenotypes in *Adamts19* mutant. Interestingly, we previously demonstrated that *Prss56* is specifically expressed in Müller cells whose cell bodies are found in the INL [13]. Together, these observations are consistent with the hypothesis of retinal *Adamts19* upregulation being part of a compensatory response to altered ocular growth. Furthermore, they suggest that Müller glia constitute a key cellular component of the ocular growth regulatory network. Interestingly, Müller glia has been postulated to act as sensors of biomechanical alterations in the retina, responding to mechanical tension by detecting subtle changes in retinal organization due to stretching of their long processes or side branches [27,28]. It is possible that impaired ocular growth (reduced ocular size) alters the structural and mechanical properties of the retina that are sensed by Müller glia triggering transcriptional activation of factors participating in the regulation of ocular axial growth [28]. Accumulating evidence indicates that mechanical cues, such as physical forces (e.g. tension, compression, or shear stress) or changes in cellular shape and size can trigger molecular responses [29]. Interestingly, the cyclic mechanical strain has been reported to induce extensive changes in the gene expression in rat Müller cells [30]. Future studies will focus on characterization of the *Prss56* promoter and the factors involved in regulating *Prss56* expression in the retina.

PRSS56 and ADAMTS19 both belong to the family of secreted serine proteases raising the possibility that they could have overlapping or redundant function(s) and act on the same substrate(s), which would explain the compensatory effect of ADAMTS19 on ocular elongation in *Prss56* deficient mice. Previous studies have reported that PRSS56 is present in the retinal inner limiting membrane (ILM) [31], which consists of Müller glia endfeet and a basement membrane rich in type IV collagen and laminins that serves as a boundary between the retina and vitreous body. It is possible that secreted PRSS56 and ADAMTS19 may promote ECM remodeling of the retinal ILM, which in turn could facilitate ocular axial growth. Notably, changes in the ILM have previously been suggested to influence ocular size [32]. Proteolytic substrates of PRSS56 and ADAMTS19 are unknown and our current efforts are focused on identifying PRSS56 substrate(s).

Using a complementary genetic approach, we show that besides promoting ocular growth during normal development, PRSS56 and MFRP are also required for pathological ocular elongation observed in a mouse model of severe and early-onset myopia caused by a null mutation in *Irbp* [23]. Indeed, inactivation of either *Prss56* or *Mfrp* prevented excessive ocular elongation in *Irpb* mutant mice, demonstrating that *Prss56* and *Mfrp* are epistatic to *Irbp* and suggesting that PRSS56 and MFRP are likely part of the same pathway modulating ocular growth. It is generally accepted that ocular elongation is driven by signals originating from the retina that are relayed to the RPE and choroid before reaching the sclera to induce scleral ECM remodeling and ocular axial growth [19,20]. The observation that inactivation of *Mfrp*, a gene expressed in the RPE and ciliary epithelium but not in the neural retina, leads to increased retinal expression of *Prss56* and *Adamts19* further suggests the existence of molecular crosstalk between the retina and the RPE in ocular axial growth [13,18]. Müller glia spans the entire length of the retina and thus, represents an ideal cell type for the transmission of retinal signals during ocular elongation. Interestingly, IRBP is primarily found in the interphotoreceptor matrix of the retina, which is located between the photoreceptor cells and the RPE [23]. It is tempting to speculate that PRSS56, IRBP, and MFRP may be part of a molecular network connecting the retina and RPE and facilitating the flow of information during ocular growth (Fig 7C). Interestingly, while no change in retinal *Adamts19* expression was detected in *Irbp* mutant mice (*Irbp*^*-/-*^*;Prss56*^*+/+*^), it was elevated in *Irbp* mutant mice that are heterozygous for mutant *Prss56 (Irbp*^*-/-*^*;Prss56*^*+/-*^) compared to control mice. This finding suggests that *Adamts19* upregulation potentially compensates for the loss of one functional *Prss56* allele and contributes to the excessive ocular elongation in *Irbp* mutant mice and further supports the notion that ADAMTS19 and PRSS56 may have an overlapping function.

The findings from the genetic mouse models used in our study are primarily relevant to ocular axial growth that is not dependent on visual experience which corresponds to prenatal and possibly postnatal vision-unadjusted ocular growth in humans. However, genetic variants of *PRSS56 and MFRP* are also associated with common forms of myopia [11,12], raising the possibility that these factors may also play a role during emmetropization. Further supporting a potential role of ADAMTS19 in postnatal ocular growth and possibly refractive development, we show that conditional ablation of *Prss56* at P21 when the eyes are exposed to visual cues [33] also causes upregulation of retinal *Adamts19* expression in the mouse. Thus, it is tempting to speculate that a molecular link between Müller glia and the RPE, mediated at least in part by PRSS56 and MFRP, serves a broader role, contributing to prenatal as well as postnatal vision-unadjusted and -adjusted ocular growth. Accordingly, any functional disruption of the molecular linkage between Müller glia and RPE could contribute to refractive errors. Furthermore, since the loss of PRSS56 or MFRP function causes a reduction in ocular axial length [6,13], it is likely that *PRSS56* and *MFRP* variants associated with myopia act via a gain of function or overexpression mechanism(s) leading to an opposite phenotype characterized by an increase in ocular axial length. Consistent with this notion, a recent study has shown that optical defocus-induced axial elongation/myopia in marmoset is accompanied by increased expression of *Prss56* [34].

Our findings raises a question as to whether the significance of *Adamts19* upregulation is limited only to severe ocular axial as observed in *PRSS56* and *MFRP* mutations or they have broader relevance in context of the axial length changes observed in general population. A recent GWAS has suggested a role for ADAMTS19 in refractive development [35]. Their findings suggest that ADAMTS19 may be part of a genetic pathway that influences both corneal curvature and ocular axial growth. Corneal curvature is closely linked to the refractive status of the eye, with flatter and steeper corneal curvature being associated with hyperopia and myopia, respectively [35]. Interestingly, although the *ADAMTS19* variant was found to influence corneal curvature and axial length, it was not associated with refractive errors. Notably, the *ADAMTS19* variant was associated with a flatter cornea and increased axial length raising the possibility that its effects on ocular elongation could overcome the hyperopia induced by a flattened cornea and suggesting a compensatory pleiotropic role for ADAMTS19 in the regulation of corneal curvature and ocular elongation during refractive development. In light of the GWAS data and findings from our mouse studies, it is tempting to speculate that human *ADAMTS19* variant acts by increasing the retinal expression of *ADAMTS19* and thereby, contributing to ocular axial length regulation in the general population.

Overall, our study identifies characteristic changes in retinal gene expression in response to impaired ocular growth and demonstrates that upregulation of retinal *Adamts19* expression is part of a compensatory molecular pathway to promote ocular elongation. Furthermore, our results highlight the critical roles of PRSS56 and MFRP in promoting ocular growth both during normal development and pathological processes underlying excessive ocular elongation in *Irbp* mutant mouse model of early-onset myopia, thus suggest a potential role for Müller glia in mediating a retina-RPE cross-talk guiding axial growth. Collectively, these findings provide important insight into the molecular mechanisms underlying ocular growth, which could have implications for the development of therapeutic interventions to prevent or slow the progression of refractive errors.

## Materials and methods

### Ethics statement

All experiments were conducted in compliance with protocols approved by the Institutional Animal Care and Use Committee at the University of California San Francisco (IACUC) (Protocols # AN181358-01D) and per the guidelines from the Association for Research in Vision and Ophthalmology’s statement on the use of animals in ophthalmic research. Animals were given access to food and water ad libitum and housed under controlled conditions including a 12-h light/dark cycle per the National Institutes of Health guidelines. Both male and female mice were used in all experiments and no differences were observed between sexes all comparisons were made between littermates to minimalize variability.

### Mouse lines

1. ***Prss56***^***-/-***^: (*C57BL/6J*.*Cg*.*Prss56*^*glcr4*^*/Sj*)- Mice carrying an ENU-induced mutation in *Prss56* causing truncation of PRSS56 protein at its C-terminal region[3].
2. ***Prss56***^***Cre/Cre***^:(*C57BL/6J*.*Cg-Prss56tm(cre)*)- Mice carrying a null *Prss56* allele in which the exon1 of *Prss56* was replaced by the CRE recombinase sequence[13,36].
3. ***Egr1***^***-/-***^: (*C57BL/6J*.*Egr1*^*tm1Jmi*^*)*- *Egr1* mutant mice generated through targeted mutation by insertion of a PGK-neo cassette introduces stop codon resulting in a null allele [37].
4. ***Adamts19***^***-/-***^: (*C57BL/6J*.*Adamts19*^*tm4a(EUCOMM)Wtsi)*^*)*- A conditional knockout mouse with *LoxP* sites flanking exon3 of *Adamts19*. Excision of the LoxP sites by the ubiquitously expressed CRE recombinase driven by beta-actin promoter leads to the generation of a knockout allele of *Adamts19* (S4A and S4B Fig) [38].

The *Adamts19* conditional mutant mice originally generated on a C57BL/6N background were backcrossed to C57BL/6J mice for a minimum of 6 generations. The *Adamts19* conditional mutant mice maintained on the original C56BL/6N background carry the retinal degeneration 8 (*rd8)* mutation in the *Crb1* gene. However, *Adamts19* mutant mice backcrossed to the C57BL/6J background used in this study do not carry the *rd8* mutation (S4C Fig).

5 ***Mfrp***^***-/-***^: *(C57BL/6J*.*C3Ga-Mfrp*^*rd6*^*)*- The mouse strain is homozygous for rd6 exhibiting retinal degeneration around four weeks during the retinal developmental phase [22].
6 ***Irbp***^***-/-***^: *(C57BL/6J*.*Irbp*^*tm1Gil*^*)*- A *Irbp* knockout mouse model [23]. This mouse line carries a targeted mutation for the *Irbp* gene, also known as *Rbp3*, where the promoter and exon 1 have been replaced by a NEO selection cassette rendering IRBP protein inactive.
7 ***Prss56***^***F/F***^: *C57BL/6J*.*Cg-Prss56tm1/Sj*, *Prss56* conditional knockout mice carrying LoxP sites flanking exons 2 to 4 of *Prss56*. Excision of exons 2–4 results in a catalytically inactive form of PRSS56 [13].
8 ***UBC-Cre ER***^***T2***^: C57BL/6J.Cg-Tg(UBC-Cre/ERT2)1Ejb, Cre-ERT2 transgenic mouse line carrying a human ubiquitin C (UBC) promoter sequence upstream of a Cre-ERT2 fusion gene.

All strains were maintained on a C57BL/6J background. PCR genotyping of all mouse strains was performed on genomic DNA obtained from tail biopsies digested with Proteinase K (Sigma, St. Louis, MO, USA) using primers listed in S2 Table. Allele-specific PCR for *Crb1* mutation (*rd8)* was done as described previously [39]. With the exceptions of a few rare cases when an eye or retina was damaged, both eyes and retina from each experimental mouse were used for SD-OCT and qPCR analyses, respectively.

### RNA-Seq analysis

Total RNA was isolated from retinas obtained from P15 *Prss56*^*-/-*^ and *Prss56*^*+/-*^ using the Qiagen RNeasy Mini Kit according to the manufacturer’s instructions. Total RNA was isolated from 3 sets of mice for each genotype (*Prss56*^*-/-*^ *or Prss56*^*+/-*^) subsequent RNA-Seq analysis (n = 3/genotype, in each set the left and right retina, were pooled). The RNA samples were submitted to the Novogene Corporation Inc. (Sacramento, USA) passed the quality control (RNA integrity number >8). A total amount of 1 μg RNA per sample was used as input material for the RNA sample preparations. Sequencing libraries were generated using NEBNextUltra RNA Library Prep Kit for Illumina (NEB, USA) following the manufacturer’s recommendations. The quality of the prepared libraries was assessed on the Agilent Bioanalyzer 2100 system. The clustering of the index-coded samples was performed on a cBot Cluster Generation System using PE Cluster Kit cBot-HS (Illumina) according to the manufacturer’s instructions. After cluster generation, the library preparations were sequenced on an Illumina platform and paired-end reads were generated. The raw FASTQ files for the RNA-Seq libraries were deposited to the NCBI Sequence Read Archive (SRA) SRP295848 and have been assigned BioProject accession PRJNA682538 along with GEO Submission (GSE162647). Outputs from the transcripts were deposited to the NCBI Gene Expression Omnibus (GEO) with accession number GSM4956165- GSM4956170. Supplemental file GSE162647_MUTANTvsCONTROL.DEG.all.txt.gz contains the genome, counts, and normalized counts used to generate the statistical plots.

### Ocular biometry

Ocular biometry was performed using Envisu R4300 spectral-domain optical coherence tomography (SD-OCT, Leica/Bioptigen Inc., Research Triangle Park, NC, USA). Measurements of various ocular parameters including axial length (AL), vitreous chamber depth (VCD), anterior chamber depth (ACD), lens diameter, and retinal thickness were performed on mice anesthetized with ketamine/xylazine (100 mg/kg and 5mg/kg, respectively; intraperitoneal (IP) following pupil dilation as described previously[13].

### Quantitative polymerase chain reaction (qPCR)

For qPCR analysis of gene expression, eyes were enucleated and retinas were immediately dissected and total RNA was extracted from mouse retinal tissue using Qiagen RNeasy Mini Kit as per manufacturers protocol (Qiagen, Valencia, CA, USA) and reverse-transcribed using iScript cDNA Synthesis Kit (Bio-Rad, Hercules, CA, USA*)* and primer sets listed in S3 Table. qPCR was performed on Bio-Rad C1000 Thermal Cycler/CF96 Real-Time System using SSOAdvanced SYBR Green Supermix (Bio-Rad, Hercules, CA, USA). Briefly, 100ng of cDNA and 10uM primers were used per reaction in a final volume of 20ul. Each cycle consisted of denaturation at 95°C for 15s, followed by annealing at 60°C for 15s, extension 72°C for 30s for a total of 39 cycles. All the experiments were run as technical duplicates and a minimum of three biological replicates were used per group. The relative expression level of each gene was normalized to housekeeping genes (*Actinβ*, *Hprt1*, and *Mapk1*) and analyzed using the CFX Maestro software (Bio-Rad, Hercules, CA, USA).

### *In situ* hybridization

Mice were transcardially perfused with ice-cold RNase-free PBS followed by 4% PFA (in RNase-free PBS). Eyes were enucleated post-fixed in RNAse-free 4% PFA, cryoprotected in 20% sucrose, and embedded in OCT compound and sectioned within 24 hours for *in situ* hybridization. QuantiGene View RNA (Affymetrix, Santa Clara, CA, USA). *In situ* hybridization was performed according to the manufacturer protocol. Briefly, 12μm cryosections were fixed overnight in 4% PFA, dehydrated through a graded series of ethanol, were subjected to 2X protease digestion for 10 minutes, fixed and hybridized with probe sets against *Adamts19* (NM_175506 (*Adamts19*), TYPE1, high sensitivity with 40~50 bp DNAs) for 3 hours at 40°C using a Thermo Brite system (Abbott Molecular, Des Plaines, IL, USA). Cryosections were then washed and subject to signal amplification and detection using a Fast Red substrate, counterstained with DAPI (blue) to visualize the nuclei, and mounted for subsequent imaging. Fluorescent images were acquired using an AxioImager M1 microscope equipped with an MRm digital camera and AxioVision software, with an LSM700 confocal microscope and Zen software (Carl Zeiss Microscopy, LLC, Germany).

### Statistical analyses

Statistical comparisons between mutant and control groups or between multiple experimental groups at a given age were performed using two-tailed unpaired Student’s t-test and one-way ANOVA, respectively, using Prism statistical software (version 6.02, GraphPad Software, San Diego, CA). A p-value < 0.05 was considered significant.

## Supporting information

S1 Fig Differential gene expression between *Prss56* mutant and control retinas. Volcano plot depicting the variance in gene expression between mutant (*Prss56*^*-/-*^) and control (*Prss56*^*+/-*^) retinas at P15. The vertical dotted line at X = 0 indicates no difference. The horizontal dotted line indicates significance for p-value adjusted (padj) = 0.05. Each point represents a gene plotted as a function of fold change (Log2 fold change, X-axis) and statistical significance (−Log 10 (p-value), Y-axis). Red: significantly upregulated differentially expressed genes (DEGs) (196); green: significantly downregulated DEGs (200).
(TIF)

S2 Fig *Prss56* and *Adamts19* expression in *Prss56*^*-/-*^ retina across ages. (**A**-**B**) Graphs showing quantification of *Prss56 (***A**) and *Adamts19* (**B**) mRNA levels using qPCR in wild-type and mutant retina at different developmental stages. A significant increase in *Adamts19* mRNA levels was detected as early as P10 in *Prss56* mutant retina (**B**), while upregulation of *Prss56* mRNA was first observed at P15 in the mutant retina (**A**). The magnitude of the increase of both *Prss56* and *Adamts19* expression became more pronounced with age in mutant retinas. *Prss56* and *Adamts19* expression were normalized to the expression of three housekeeping genes (*Hprt1*, *Actb1*, and *Mapk1)*. Data are presented as fold expression relative to wild-type (mean ± SEM), N = 4 to 6 retinas/group. *p<0.05; **p<0.01; ***p<0.001, t-test. The *Prss56* qPCR data shown in **A** was previously published in Fig 4 of Paylakhi et al. 2018 [13] and is shown here to facilitate direct comparison between the relative levels of *Prss56* and *Adamts19* as throughout development.
(TIF)

S3 Fig *Egr1* inactivation normalizes the retinal expression of *Prss56* and *Adamts19* expression in *Prss56* mutant mice. (**A**-**B**) Graphs showing quantification of *Prss56 (***A**) and *Adamts19* (**B**) mRNA levels using qPCR analysis in retina from P35 mice. While no difference in retinal *Prss56* and *Adamts19* expression was observed between *Egr1* mutant (*Egr1*^*-/-*^*; Prss56*^*+/-*^) and control *Egr1*^*+/+*^*; Prss56*^*+/-*^ mice, *Prss56* and *Adamst19* mRNA levels were significantly increased in *Prss56* mutant mice (*Egr1*^*+/+*^*; Prss56*^*-/-*^ and *Egr1*^*+/-*^*;Prss56*^*-/-*^). Importantly, *Egr1* inactivation reduced the expression of *Prss56* and *Adamts19* in *Prss56* mutant retina to levels comparable to those detected in controls retina (compare *Egr1*^*-/-*^*; Prss56*^*-/-*^ to *Egr1*^*+/+*^*;Prss56*^*+/-*^). *Prss56* and *Adamts19* expression were normalized to the expression of three housekeeping genes (*Hprt1*, *Actb1*, and *Mapk1)*. Data are presented as fold expression relative to control *Egr1*^*+/+*^*;Prss56*^*+/-*^ retina (mean ± SEM), N = 6 retinas /group. ***p<0.001, ****p<0.0001 (for comparison to control *Egr1*^*+/+*^;*Prss56*^*+/-*^ mice), one-way ANOVA.
(TIF)

S4 Fig Generation of *Adamts19* mutant mice. (**A**) The *LoxP* sites (red triangles) flank the exon 3 of the *Adamts19*^*F*^ allele. In presence of Cre recombinase, exon 3 is deleted resulting in a frameshift mutation and premature stop codon, rendering ADAMTS19 catalytically inactive. (**B)** PCR amplification of DNA from wild-type (*Adamts19*^*+/+*^, lane 1), heterozygous (*Adamts19*^*F/+*,^ lane 2) or homozygous (*Adamts19*^*F/F*^) mice. PCR reactions were performed using primers amplifying a region of exon 3 (F1+R1). Deletion of exon 3 from the *Adamts19*^*F/F*^ allele was confirmed by the absence of a PCR product. (**C**) PCR analysis showing that *Adamts19* mutant mice (F/F) maintained on a C57BL/6N (B6N) but not on a C57BL/6J (B6J) background carry the *rd8* mutation at the *Crb1* locus. (**D**) qPCR analysis using primer sets to amplify exon 3 (Ex3) or a region contained between the end of exon 2 and beginning of exon 4 (Ex2,4) confirming excision of exon 3 (Ex3) and showing the absence of *Adamts19* RNA decay (Ex2,4) in *Adamts19*^*F/F*^ retina following Cre-mediated recombination for the generation of *Adamts19*^*-/-*^ mice (compare *Adamts19*^*-/-*^ to *Adamts19*^*+/+*^).
(TIF)

S5 Fig The *Adamts19* mutant allele gives rise to a catalytically inactive truncated variant. (**A-C**) Schematic diagram (**A**) and amino acid sequences of full-length wild-type (**B**) and mutant (**C**) ADAMTS19 protein showing that the *Adamts19* mutation leads to a null allele by causing a frameshift mutation and premature stop codon, leading to a truncated and catalytically inactive protein.
(TIF)

S6 Fig Ocular biometric analysis in *Adamts19* mutant mice. Histograms showing that the lens diameter (**A**) and retinal thickness (**B**) were indistinguishable between *Adamts19*^*-/-*^, *Adamts19*^*+/-*^ and control *Adamts19*^*+/+*^ mice. Data are presented as mean ± SD, N≥7 eyes/group.
(TIF)

S7 Fig Ocular biometric parameters are indistinguishable between *Adamts19*^*+/-*^*;Prss56*^*+/-*^ and wild-type mice. (**A-D**) Histograms showing that all the ocular biometric parameters examined including axial length (**A**) VCD (**B**), lens diameters (**C**), and retinal thickness (**D**) are indistinguishable between wild-type (*Adamts19*^*+/+*^*;Prss56*^*+/+*^) and *Adamts19*^*+/-*^*;Prss56*^*+/-*^ mice at P18. Data are presented as mean ± SD, N≥7 eyes/group.
(TIF)

S8 Fig Ocular biometric analysis of *Adamts19*^*-/-*^*;Prss56*^*-/-*^ mice. (**A-B**) Histograms showing quantification of the lens diameter (**A**) and retinal thickness (**B**). (**A**) The lens diameter is indistinguishable between *Adamts19*^*+/+*^*;Prss56*^*-/-*^, *Adamts19*^*+/-*^*;Prss56*^*-/-*^, and *Adamts19*^*-/-*^*;Prss56*^*-/-*^ mice at both ages examined (P18 and P30). (**B**) Retinal thickness was significantly increased in all three groups of mice deficient for *Prss56* compared to the controls (*Adamts19*^*+/-*^*; Prss56*^*+/-*^). The increase in retinal thickness observed in *Prss56* deficient mice was exacerbated by *Adamts19* inactivation at P18 but not P30. Data are presented as mean ± SD, N≥13 eyes/group. ***p<0.001 (for comparison to control *Adamts19*^*+/-*^;*Prss56*^*+/-*^ retina); ^‡^p<0.05 (for comparison to *Adamts19*^*-/-*^;*Prss56*^*-/-*^ retina), one-way ANOVA. (**C**) Graph showing the relative expression of *Adamts19* using qPCR in *Adamts19*^*+/-*^*;Prss56*^*+/-*^, *Adamts19*^*+/+*^*;Prss56*^*-/-*^ and *Adamts19*^*+/-*^*;Prss56*^*-/-*^ retina. A significant increase in *Adamts19* mRNA levels was detected in *Prss56* deficient mice *(Adamts19*^*+/+*^*;Prss56*^*-/-*^ and *Adamts19*^*+/-*^*;Prss56*^*-/-*^*)* compared to control *Adamts19*^*+/-*^*;Prss56*^*+/-*^ littermates (P18). Notably, *Adamts19* expression was significantly higher in *Prss56* mutant mice carrying two wild-type *Adamts19* alleles *(Adamts19*^*+/+*^*;Prss56*^-/-^) compared to those that are heterozygous for the *Adamts19* mutant allele *(Adamts19*^*+/-*^*;Prss56*^-/-^). Data are presented as fold relative to control retina (mean ± SD), N≥6 retinas/group. **p<0.01, ^**‡‡**^p<0.01, ****p<0.0001 one-way ANOVA.
(TIF)

S9 Fig Ocular biometric parameters are indistinguishable between *Adamts19*^*+/-*^*;Mfrp*^*+/-*^ and wild-type mice. (**A-D**) Histograms showing that all the ocular biometric parameters examined, including axial length (**A**), VCD (**B**), lens diameter (**C**), and retinal thickness (**D**) are indistinguishable between wild-type (*Adamts19*^*+/+*^*;Mfrp*^*+/+*^) and *Adamts19*^*+/-*^*;Mfrp*^*+/-*^ mice at P18. Data are presented as mean ± SD, N≥8 eyes/group.
(TIF)

S10 Fig Ocular biometric analysis of *Adamts19*^*-/-*^*;Mfrp*^*-/-*^ mice. (**A-C**) Histograms showing that the lens diameter (**A**) is indistinguishable between *Adamts19*^*+/-*^;*Mfrp*^*-/-*^, *Adamts19*^*-/-*^;*Mfrp*^*-/-*^, and control *(Adamts19*^*+/-*^;*Mfrp*^*+/-*^) mice, while retinal thickness (**B**) is increased in both *Adamts19*^*+/-*^;*Mfrp*^*-/-*^ and *Adamts19*^*-/-*^;*Mfrp*^*-/-*^ mice compared to control *Adamts19*^*+/-*^;*Mfrp*^*+/-*^ mice at P18. Data are presented as mean ± SD, N≥11eyes/group. ****p<0.0001, one-way ANOVA.
(TIF)

S11 Fig Ocular biometric parameters are indistinguishable between *Irbp*^*+/-*^*;Prss56*^*+/-*^ or *Irbp*^*+/-*^*;Mfrp*^*+/-*^ and wild-type mice. (**A-H**) Histograms showing that all the ocular biometric parameters examined, including axial length (**A, E**), VCD (**B, F**), lens diameter (**C, G**), and retinal thickness (**D, H**) are indistinguishable between wild-type and *Irbp*^*+/-*^*;Prss56*^*+/-*^ or *Irbp*^*+/-*^*;Mfrp*^*+/-*^ mice at P18. Data are presented as mean ± SD, N≥8 eyes/group.
(TIF)

S12 Fig Ocular biometric analysis of *Irbp* mutant mice following *Prss56* inactivation. (**A**) Representative OCT images showing that ocular axial length (quantified in Fig 6A) and VCD (quantified in Fig 6B) are increased in *Irbp* mutant mice (*Irbp*^*-/-*^*;Prss56*^*+/-*^) and reduced in *Prss56* mutant mice compared to control *Irbp*^*+/-*^*;Prss56*^*+/-*^ control mice (data from P18 mice are shown). (**B**) In contrast, retinal thickness was reduced in *Irbp* mutant mice (*Irbp*^*-/-*^*;Prss56*^*+/-*^) and increased in *Prss56* mutant and *Irbp;Prss56* double mutant mice (*Irbp*^*+/-*^*;Prss56*^*-/-*^ and *Irbp*^*-/-*^*;Prss56*^*-/-*^, respectively) compared to control *Irbp*^*+/-*^*; Prss56*^*+/-*^ mice. Data are presented as mean ± SD, N≥4 eyes/group. *p<0.05; ***p<0.0001 (compared to *Irbp*^*+/-*^*; Prss56*^*+/-*^ controls); ^‡‡‡^p<0.0001 (compared to *Irbp*^*-/-*^;*Prss56*^*-/-*^ mice), one-way ANOVA.
(TIF)

S13 Fig Ocular biometric analysis of *Irbp* mutant mice following *Mfrp* inactivation. (**A**) Representative OCT images showing that ocular axial length (quantified in Fig 6C) and VCD (quantified in Fig 6D) are increased in *Irbp* mutant mice (*Irbp*^*-/-*^*;Mfrp*^*+/-*^) and reduced in *Mfrp* mutant and *Irbp;Mfrp* double mutant mice (*Irbp*^*+/-*^*;Mfrp*^*-/-*^ and *Irbp*^*-/-*^*;Mfrp*^*-/-*^, respectively) compared to control *Irbp*^*+/-*^*;Mfrp*^*+/-*^ mice at P18. (**B**) In contrast, and retinal thickness was reduced in *Irbp* mutant mice (*Irbp*^*-/-*^*; Mfrp*^*+/-*^) and increased in *Mfrp* mutant and *Irbp;Mfrp* double mutant mice (*Irbp*^*+/-*^*;Mfrp*^*-/-*^ and *Irbp*^*-/-*^*;Mfrp*^*-/-*^, respectively) compared to control *Irbp*^*+/-*^*; Mfrp*^*+/-*^ mice. Data are presented as mean ± SD, N≥8 eyes/group. ***p<0.0001 (compared to *Irbp*^*+/-*^*; Mfrp*^*+/-*^ controls); ^**‡‡‡**^p<0.0001 (compared to *Irbp*^*-/-*^; *Mfrp*^*-/-*^ mice), one-way ANOVA.
(TIF)

S14 Fig *Adamts19* expression in *Irbp* mutant and *Irbp;Prss56 mutant* mice. Graphs showing quantification of *Adamts19* mRNA levels using qPCR analysis in the retina from P18 mice. *Adamts19* expression was indistinguishable between wild-type (*Irbp*^*+/+*^*;Prss56*^*+/+*^), double heterozygous (*Irbp*^*+/-*^*; Prss56*^*+/-*^), and *Irbp* mutant mice carrying two wild-type alleles of *Prss56* (*Irbp*^*-/-*^*; Prss56*^*+/+*^). In contrast, upregulation of retinal *Adamts19* expression was observed in *Irbp* mutant mice that are heterozygous for *Prss56* mutant allele (*Irbp*^*-/-*^*;Prss56*^*+/-*^). As expected, *Adamts19* expression was significantly upregulated in *Prss56* single mutant mice (*Irbp*^*+/-*^*; Prss56*^*-/-*^) compared to *Irbp*^*+/+*^*;Prss56*^*+/+*^ and *Irbp*^*+/-*^*;Prss56*^*+/-*^ control mice. Interestingly, *Adamts19* expression was further elevated in double mutant mice (*Irbp*^*-/-*^*; Prss56*^*-/-*^) compared to *Prss56* single mutant mice (*Irbp*^*+/-*^*; Prss56*^*-/-*^). Data are presented as fold expression relative to control *Irbp*^*+/+*^*;Prss56*^*+/+*^ retina (mean ± SEM), N≥4 retinas /group. ***p<0.001; ****p<0.0001 (compared to control *Irbp*^*+/-*^*; Prss56*^*+/-*^ mice); ^**‡‡‡‡**^p<0.0005 (compared to *Irbp*^*-/-*^; *Prss56*^*-/-*^ mice), one-way ANOVA.
(TIF)

S1 Table List of genes differentially expressed between *Prss56*^*-/-*^ and control *Prss56*^*+/-*^ retina. (XLSX)

S2 Table List of genotyping primers. (XLSX)

S3 Table List of qPCR primers. (XLSX)

S1 Data Complete list of differentially expressed genes between *Prss56*^*-/-*^ and control *Prss56*^*+/-*^ retina. (XLSX)
