# Supplementary figures and images for: Identification of MFRP and the secreted serine proteases PRSS56 and ADAMTS19 as part of a molecular network involved in ocular growth regulation
Source: PLoS Genet. 2021 Mar 23;17(3):e1009458. doi: 10.1371/journal.pgen.1009458 (PMC8018652; doi:10.1371/journal.pgen.1009458)

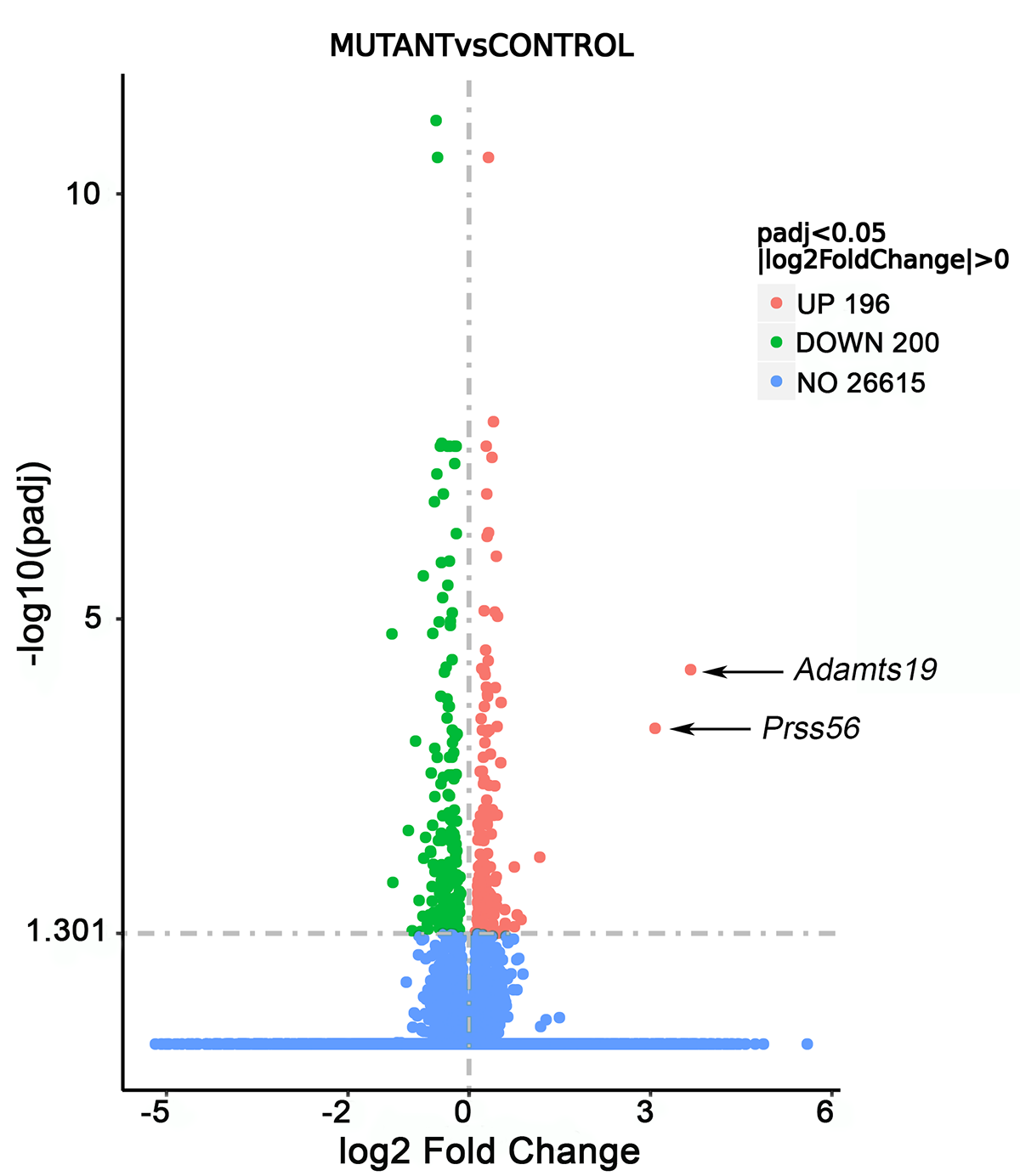

Supplement: S1 Fig — Volcano plot depicting the variance in gene expression between mutant (Prss56-/-) and control (Prss56+/-) retinas at P15. The vertical dotted line at X = 0 indicates no difference. The horizontal dotted line indicates significance for p-value adjusted (padj) = 0.05. Each point represents a gene plotted as a function of fold change (Log2 fold change, X-axis) and statistical significance (−Log 10 (p-value), Y-axis). Red: significantly upregulated differentially expressed genes (DEGs) (196); green: significantly downregulated DEGs (200). (TIF) [file pgen.1009458.s001.tif]

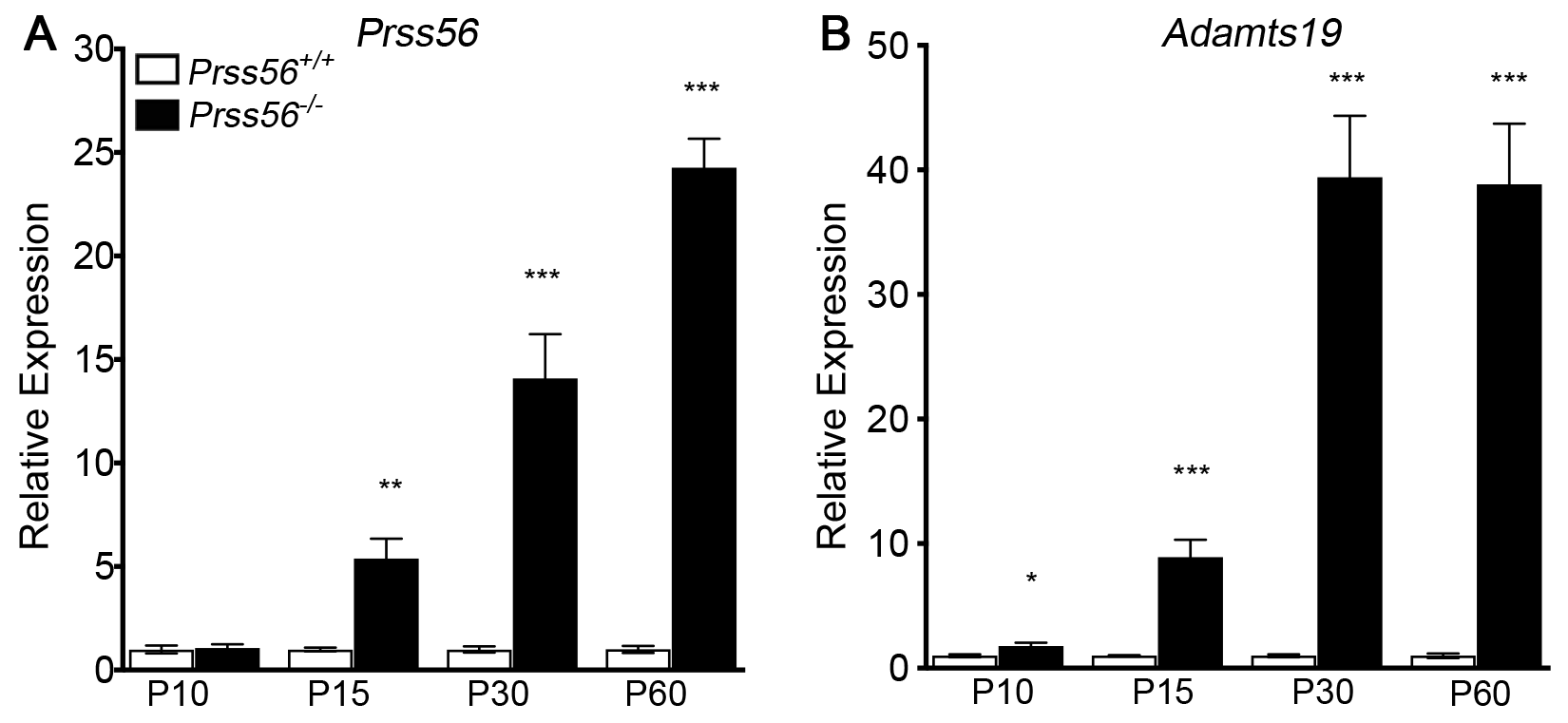

Supplement: S2 Fig — (A-B) Graphs showing quantification of Prss56 (A) and Adamts19 (B) mRNA levels using qPCR in wild-type and mutant retina at different developmental stages. A significant increase in Adamts19 mRNA levels was detected as early as P10 in Prss56 mutant retina (B), while upregulation of Prss56 mRNA was first observed at P15 in the mutant retina (A). The magnitude of the increase of both Prss56 and Adamts19 expression became more pronounced with age in mutant retinas. Prss56 and Adamts19 expression were normalized to the expression of three housekeeping genes (Hprt1, Actb1, and Mapk1). Data are presented as fold expression relative to wild-type (mean ± SEM), N = 4 to 6 retinas/group. *p<0.05; **p<0.01; ***p<0.001, t-test. The Prss56 qPCR data shown in A was previously published in Fig 4 of Paylakhi et al. 2018 [13] and is shown here to facilitate direct comparison between the relative levels of Prss56 and Adamts19 as throughout development. (TIF) [file pgen.1009458.s002.tif]

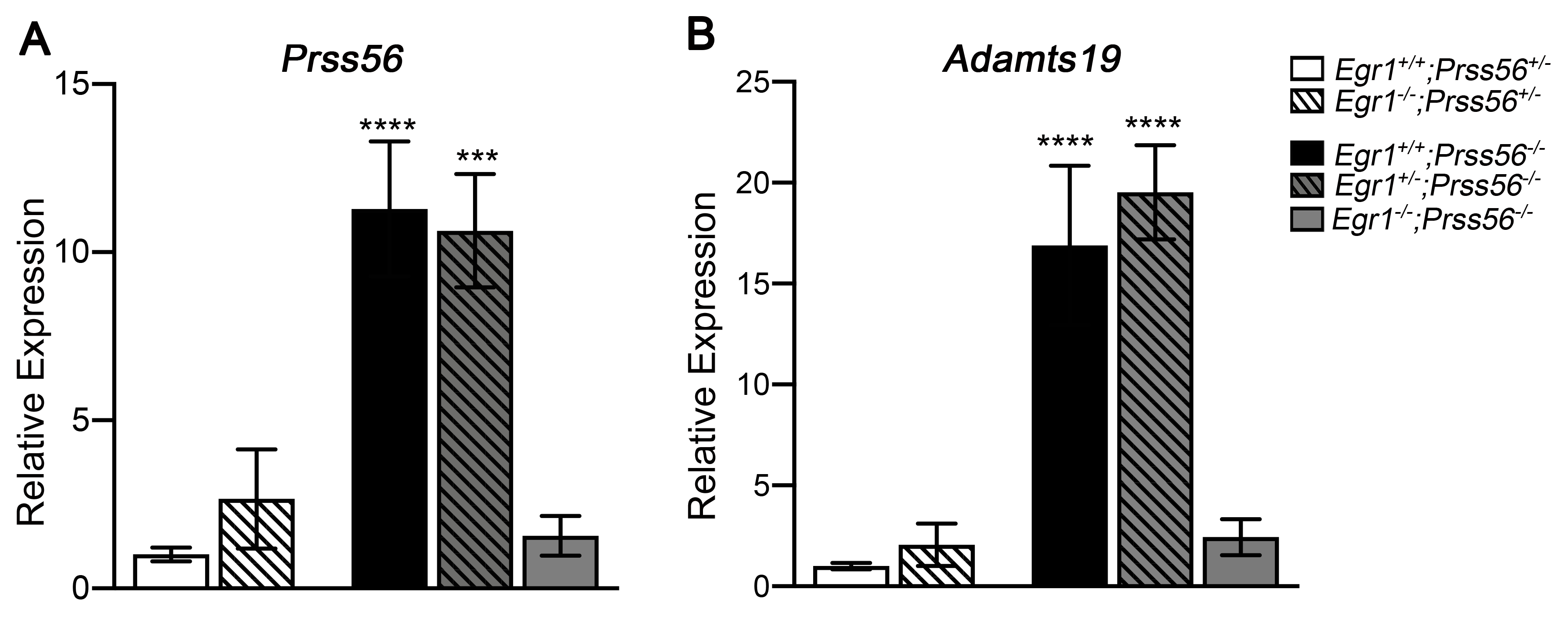

Supplement: S3 Fig — (A-B) Graphs showing quantification of Prss56 (A) and Adamts19 (B) mRNA levels using qPCR analysis in retina from P35 mice. While no difference in retinal Prss56 and Adamts19 expression was observed between Egr1 mutant (Egr1-/-; Prss56+/-) and control Egr1+/+; Prss56+/- mice, Prss56 and Adamst19 mRNA levels were significantly increased in Prss56 mutant mice (Egr1+/+; Prss56-/- and Egr1+/-;Prss56-/-). Importantly, Egr1 inactivation reduced the expression of Prss56 and Adamts19 in Prss56 mutant retina to levels comparable to those detected in controls retina (compare Egr1-/-; Prss56-/- to Egr1+/+;Prss56+/-). Prss56 and Adamts19 expression were normalized to the expression of three housekeeping genes (Hprt1, Actb1, and Mapk1). Data are presented as fold expression relative to control Egr1+/+;Prss56+/- retina (mean ± SEM), N = 6 retinas /group. ***p<0.001, ****p<0.0001 (for comparison to control Egr1+/+;Prss56+/- mice), one-way ANOVA. (TIF) [file pgen.1009458.s003.tif]

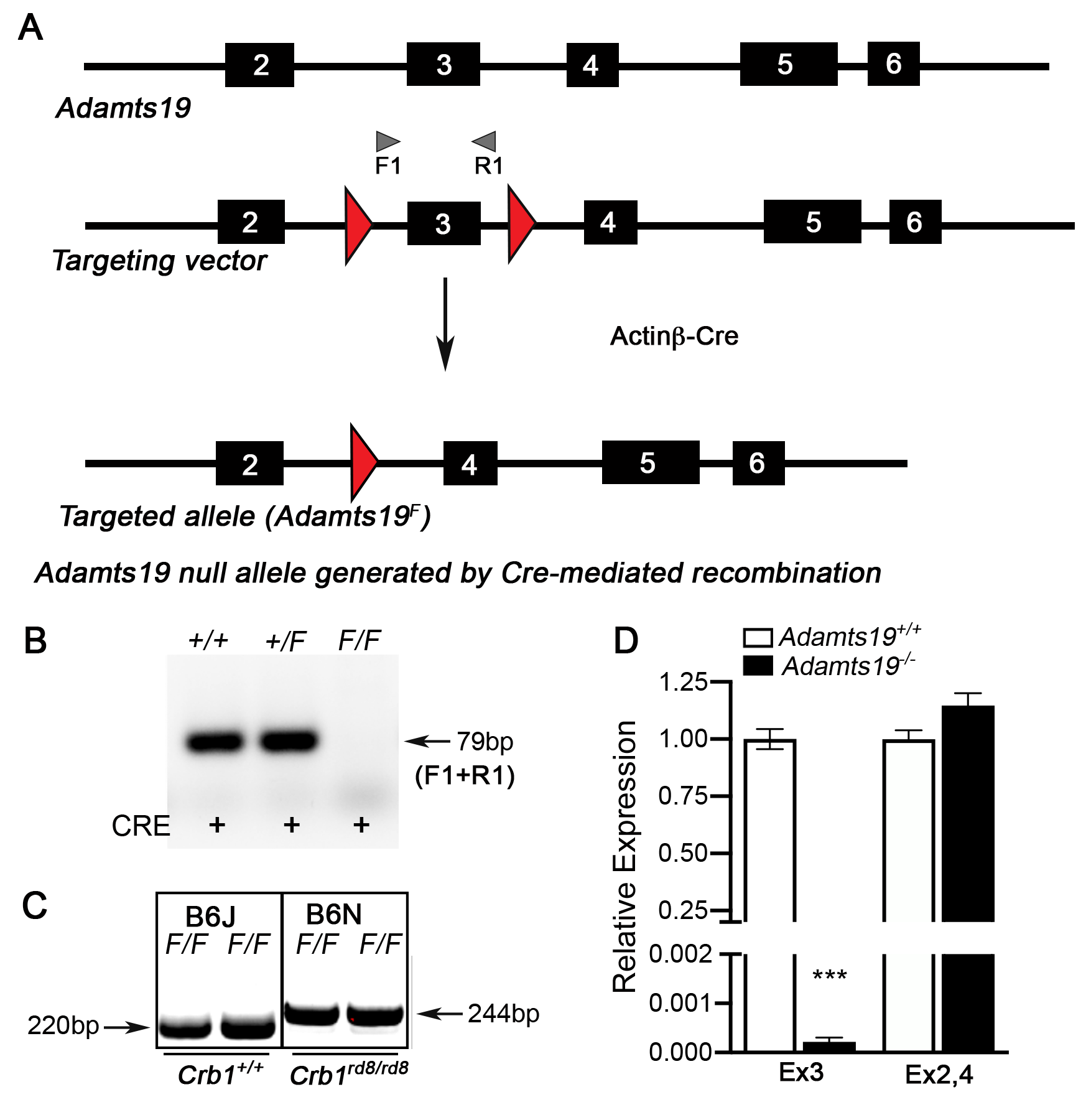

Supplement: S4 Fig — (A) The LoxP sites (red triangles) flank the exon 3 of the Adamts19F allele. In presence of Cre recombinase, exon 3 is deleted resulting in a frameshift mutation and premature stop codon, rendering ADAMTS19 catalytically inactive. (B) PCR amplification of DNA from wild-type (Adamts19+/+, lane 1), heterozygous (Adamts19F/+, lane 2) or homozygous (Adamts19F/F) mice. PCR reactions were performed using primers amplifying a region of exon 3 (F1+R1). Deletion of exon 3 from the Adamts19F/F allele was confirmed by the absence of a PCR product. (C) PCR analysis showing that Adamts19 mutant mice (F/F) maintained on a C57BL/6N (B6N) but not on a C57BL/6J (B6J) background carry the rd8 mutation at the Crb1 locus. (D) qPCR analysis using primer sets to amplify exon 3 (Ex3) or a region contained between the end of exon 2 and beginning of exon 4 (Ex2,4) confirming excision of exon 3 (Ex3) and showing the absence of Adamts19 RNA decay (Ex2,4) in Adamts19F/F retina following Cre-mediated recombination for the generation of Adamts19-/- mice (compare Adamts19-/- to Adamts19+/+). (TIF) [file pgen.1009458.s004.tif]

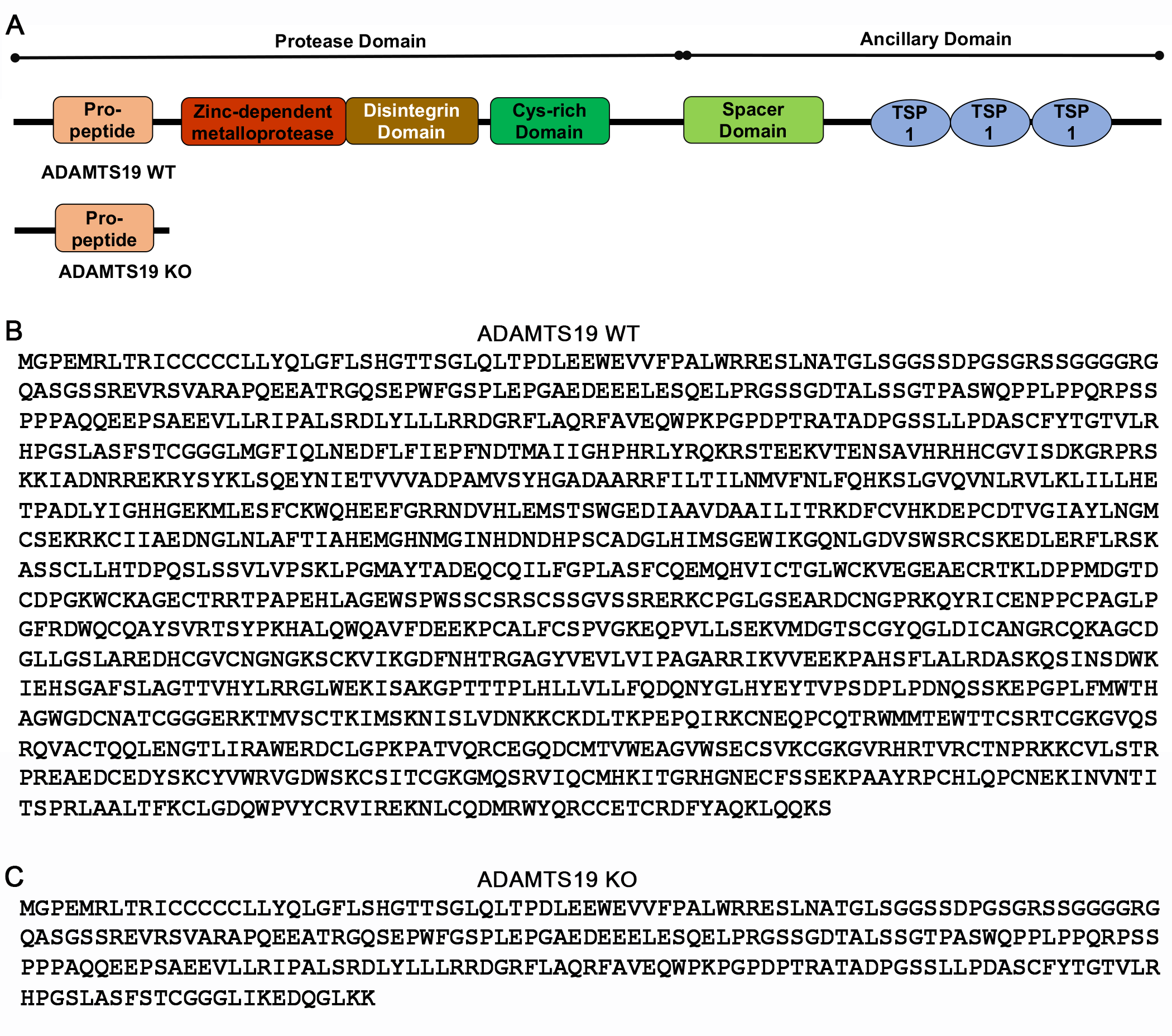

Supplement: S5 Fig — (A-C) Schematic diagram (A) and amino acid sequences of full-length wild-type (B) and mutant (C) ADAMTS19 protein showing that the Adamts19 mutation leads to a null allele by causing a frameshift mutation and premature stop codon, leading to a truncated and catalytically inactive protein. (TIF) [file pgen.1009458.s005.tif]

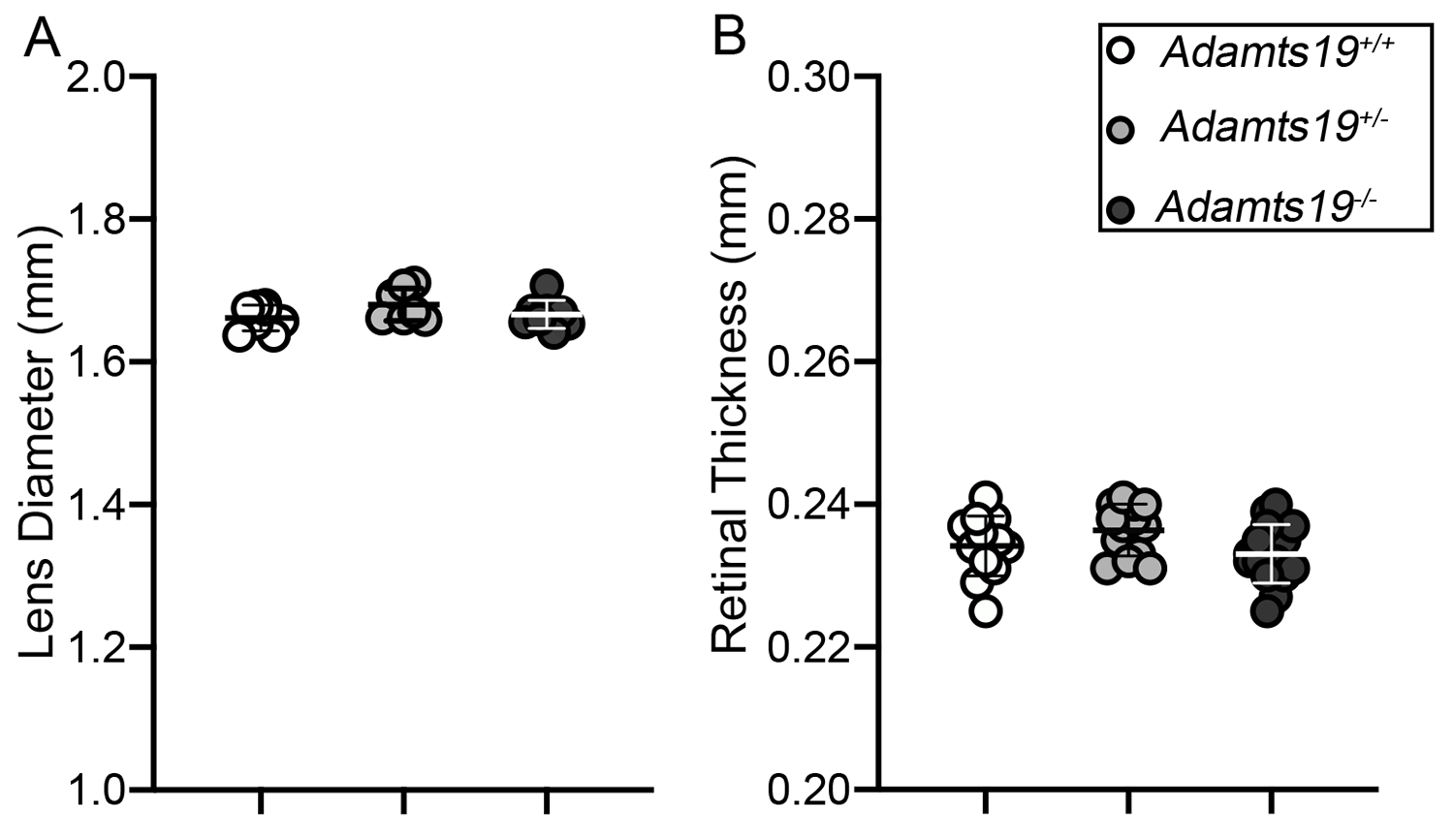

Supplement: S6 Fig — Histograms showing that the lens diameter (A) and retinal thickness (B) were indistinguishable between Adamts19-/-, Adamts19+/- and control Adamts19+/+ mice. Data are presented as mean ± SD, N≥7 eyes/group. (TIF) [file pgen.1009458.s006.tif]

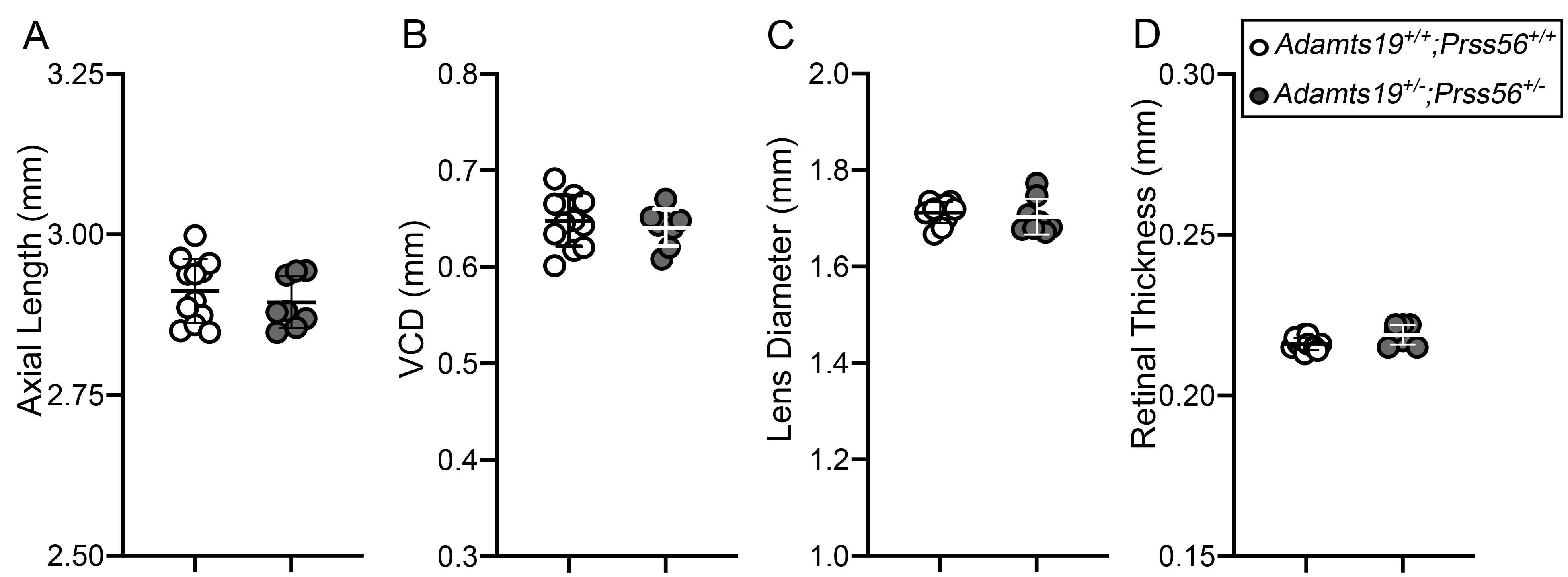

Supplement: S7 Fig — (A-D) Histograms showing that all the ocular biometric parameters examined including axial length (A) VCD (B), lens diameters (C), and retinal thickness (D) are indistinguishable between wild-type (Adamts19+/+;Prss56+/+) and Adamts19+/-;Prss56+/- mice at P18. Data are presented as mean ± SD, N≥7 eyes/group. (TIF) [file pgen.1009458.s007.tif]

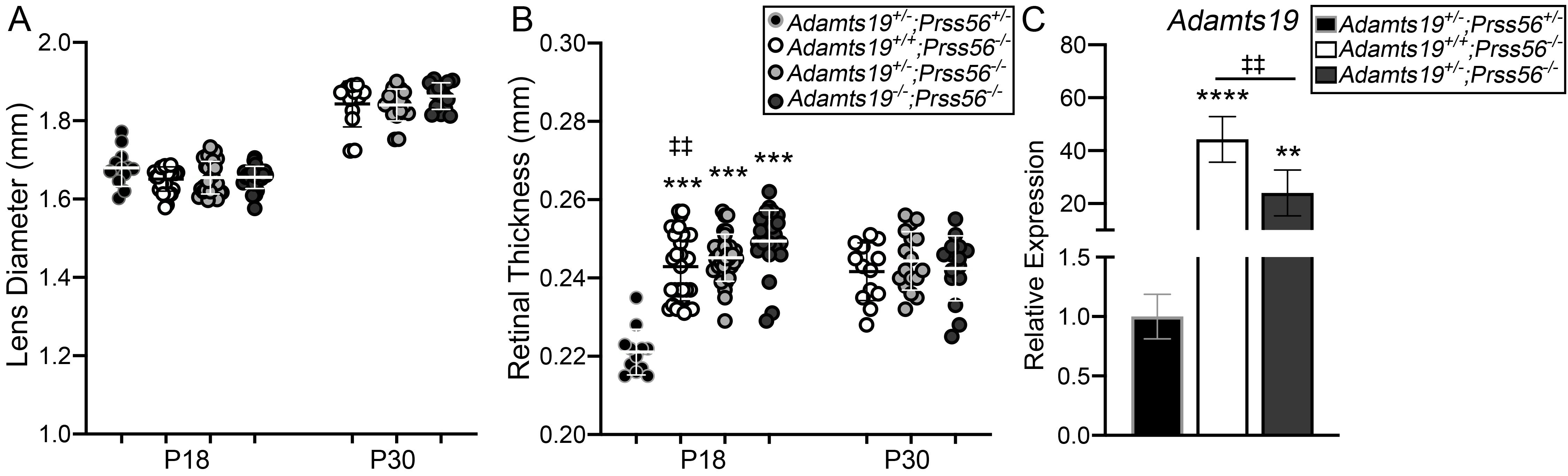

Supplement: S8 Fig — (A-B) Histograms showing quantification of the lens diameter (A) and retinal thickness (B). (A) The lens diameter is indistinguishable between Adamts19+/+;Prss56-/-, Adamts19+/-;Prss56-/-, and Adamts19-/-;Prss56-/- mice at both ages examined (P18 and P30). (B) Retinal thickness was significantly increased in all three groups of mice deficient for Prss56 compared to the controls (Adamts19+/-; Prss56+/-). The increase in retinal thickness observed in Prss56 deficient mice was exacerbated by Adamts19 inactivation at P18 but not P30. Data are presented as mean ± SD, N≥13 eyes/group. ***p<0.001 (for comparison to control Adamts19+/-;Prss56+/- retina); ‡p<0.05 (for comparison to Adamts19-/-;Prss56-/- retina), one-way ANOVA. (C) Graph showing the relative expression of Adamts19 using qPCR in Adamts19+/-;Prss56+/-, Adamts19+/+;Prss56-/- and Adamts19+/-;Prss56-/- retina. A significant increase in Adamts19 mRNA levels was detected in Prss56 deficient mice (Adamts19+/+;Prss56-/- and Adamts19+/-;Prss56-/-) compared to control Adamts19+/-;Prss56+/- littermates (P18). Notably, Adamts19 expression was significantly higher in Prss56 mutant mice carrying two wild-type Adamts19 alleles (Adamts19+/+;Prss56-/-) compared to those that are heterozygous for the Adamts19 mutant allele (Adamts19+/-;Prss56-/-). Data are presented as fold relative to control retina (mean ± SD), N≥6 retinas/group. **p<0.01, ‡‡p<0.01, ****p<0.0001 one-way ANOVA. (TIF) [file pgen.1009458.s008.tif]

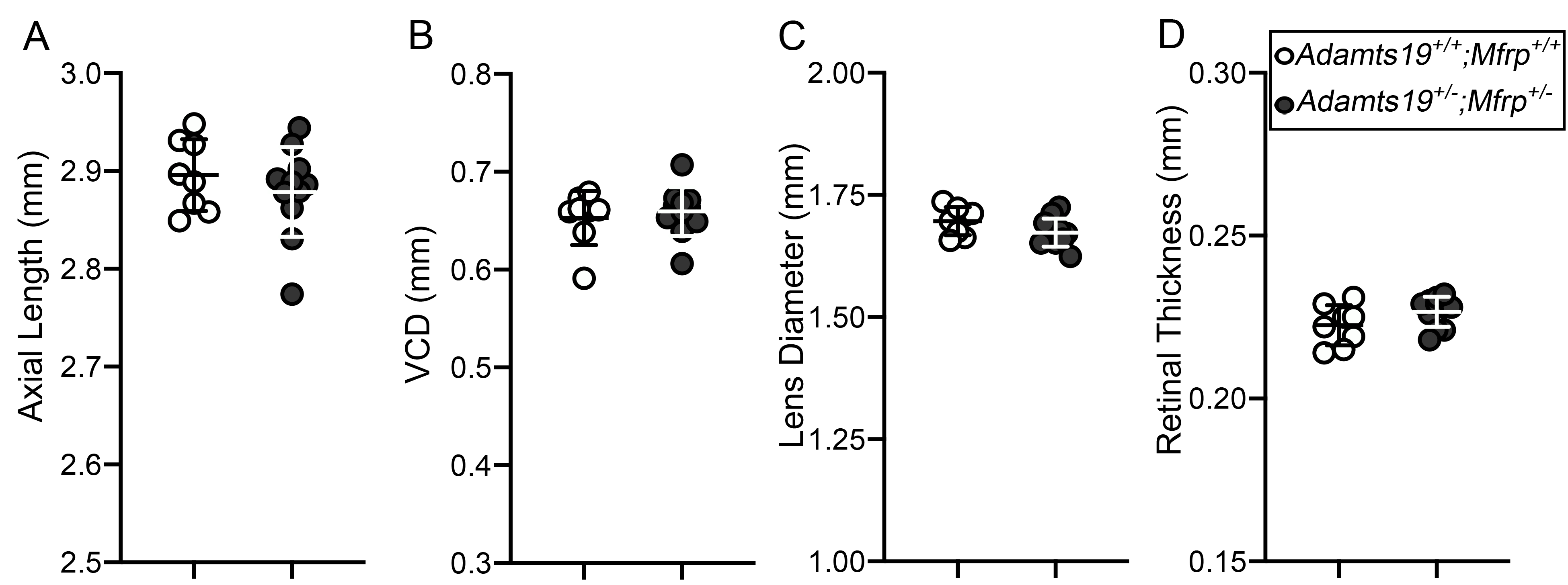

Supplement: S9 Fig — (A-D) Histograms showing that all the ocular biometric parameters examined, including axial length (A), VCD (B), lens diameter (C), and retinal thickness (D) are indistinguishable between wild-type (Adamts19+/+;Mfrp+/+) and Adamts19+/-;Mfrp+/- mice at P18. Data are presented as mean ± SD, N≥8 eyes/group. (TIF) [file pgen.1009458.s009.tif]

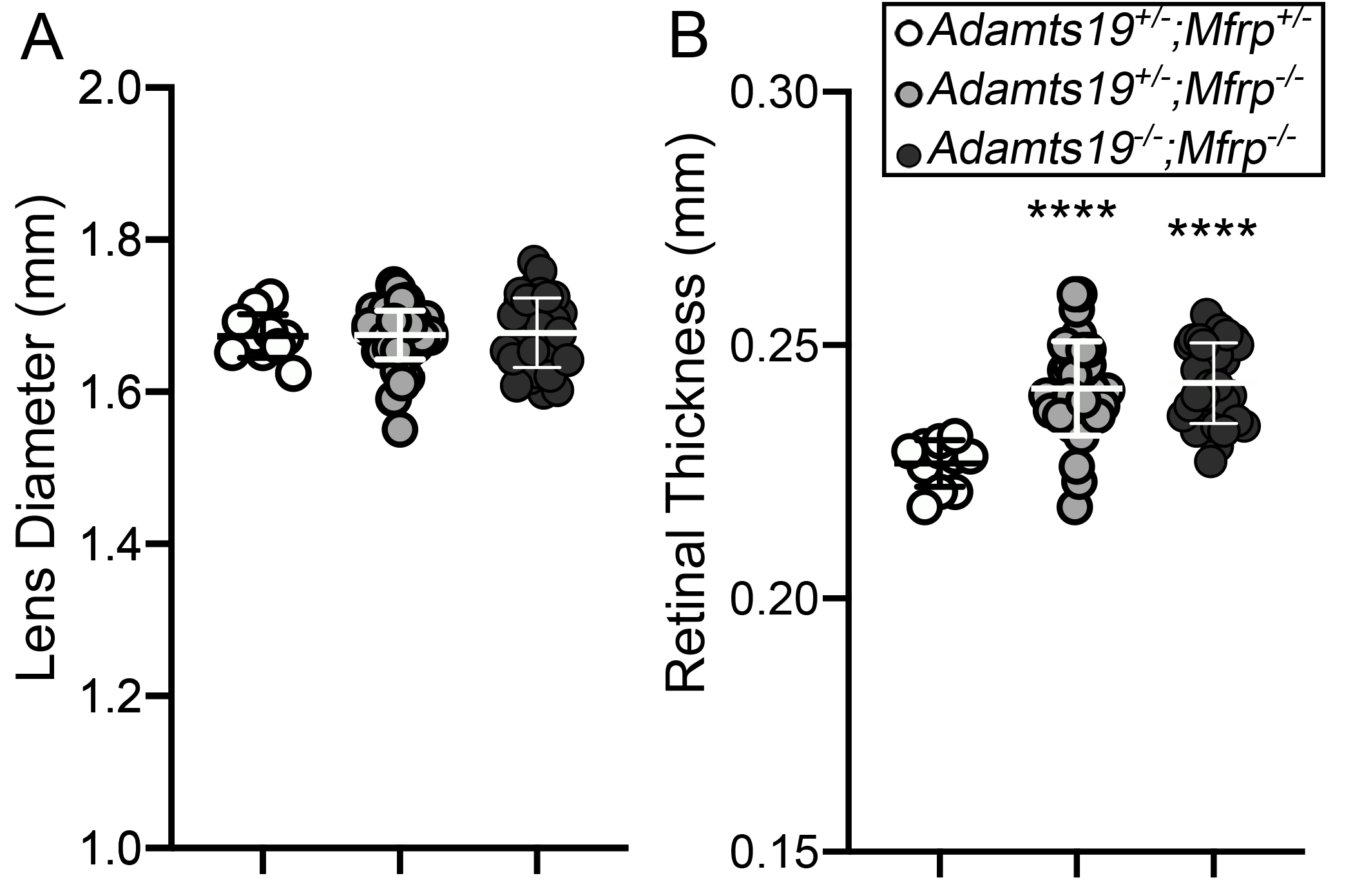

Supplement: S10 Fig — (A-C) Histograms showing that the lens diameter (A) is indistinguishable between Adamts19+/-;Mfrp-/-, Adamts19-/-;Mfrp-/-, and control (Adamts19+/-;Mfrp+/-) mice, while retinal thickness (B) is increased in both Adamts19+/-;Mfrp-/- and Adamts19-/-;Mfrp-/- mice compared to control Adamts19+/-;Mfrp+/- mice at P18. Data are presented as mean ± SD, N≥11eyes/group. ****p<0.0001, one-way ANOVA. (TIF) [file pgen.1009458.s010.tif]

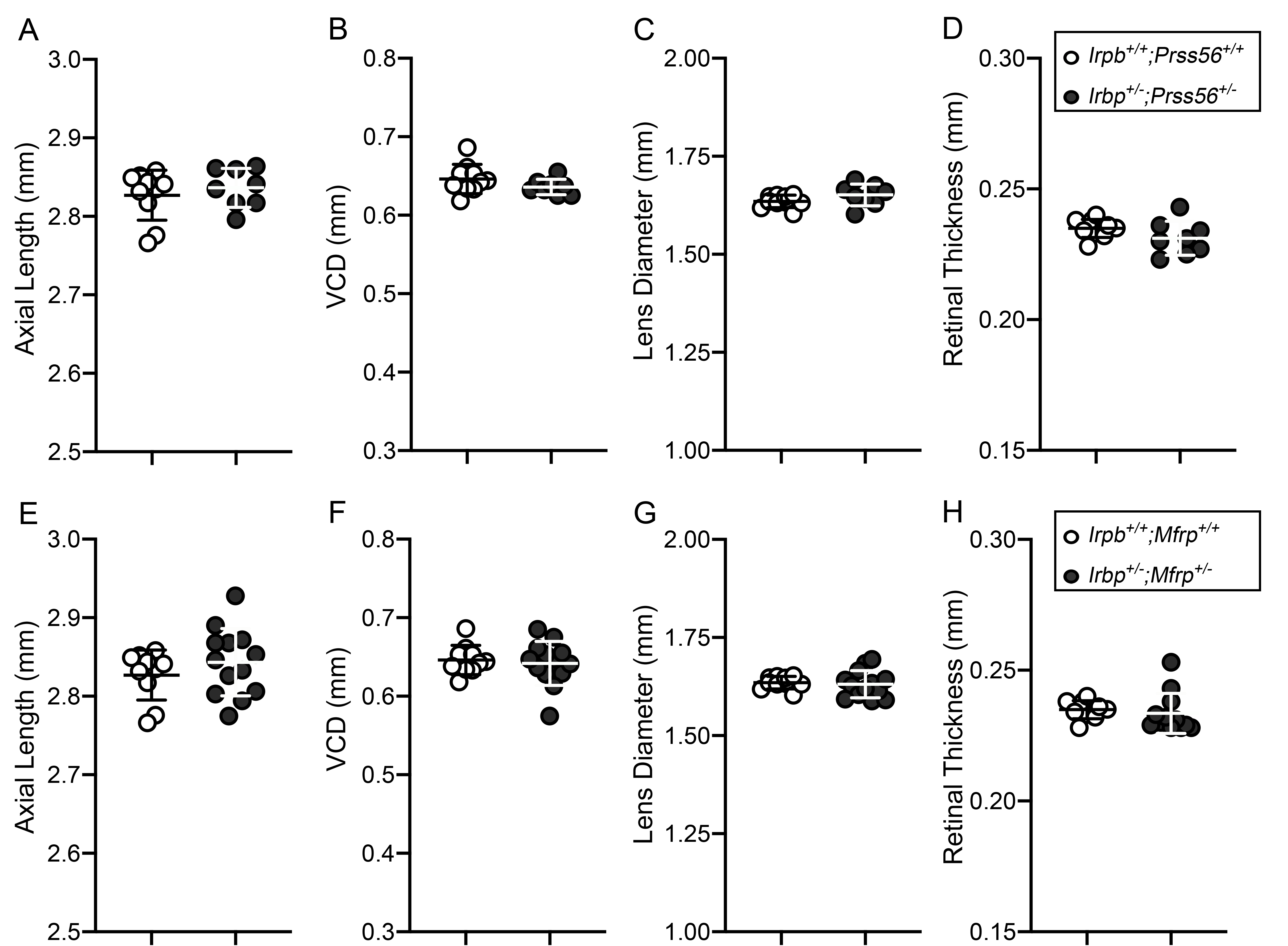

Supplement: S11 Fig — (A-H) Histograms showing that all the ocular biometric parameters examined, including axial length (A, E), VCD (B, F), lens diameter (C, G), and retinal thickness (D, H) are indistinguishable between wild-type and Irbp+/-;Prss56+/- or Irbp+/-;Mfrp+/- mice at P18. Data are presented as mean ± SD, N≥8 eyes/group. (TIF) [file pgen.1009458.s011.tif]

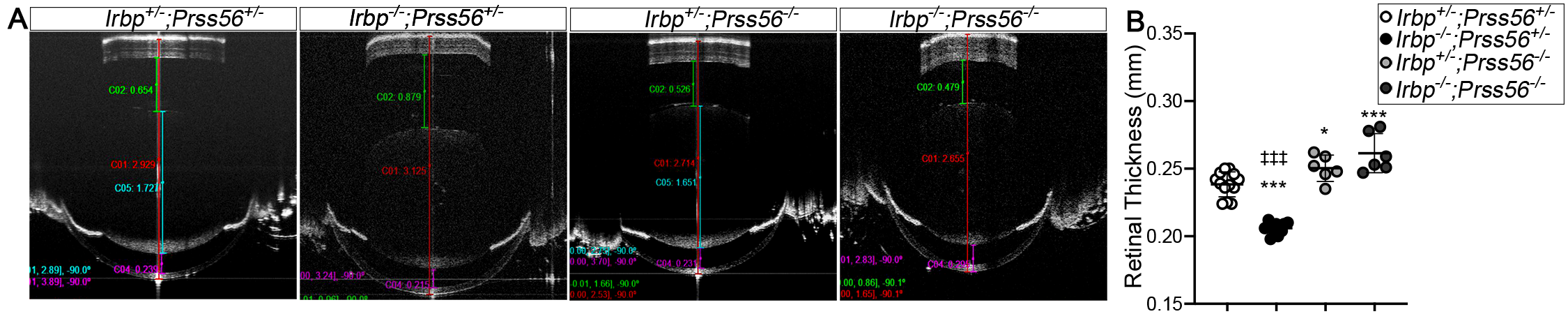

Supplement: S12 Fig — (A) Representative OCT images showing that ocular axial length (quantified in Fig 6A) and VCD (quantified in Fig 6B) are increased in Irbp mutant mice (Irbp-/-;Prss56+/-) and reduced in Prss56 mutant mice compared to control Irbp+/-;Prss56+/- control mice (data from P18 mice are shown). (B) In contrast, retinal thickness was reduced in Irbp mutant mice (Irbp-/-;Prss56+/-) and increased in Prss56 mutant and Irbp;Prss56 double mutant mice (Irbp+/-;Prss56-/- and Irbp-/-;Prss56-/-, respectively) compared to control Irbp+/-; Prss56+/- mice. Data are presented as mean ± SD, N≥4 eyes/group. *p<0.05; ***p<0.0001 (compared to Irbp+/-; Prss56+/- controls); ‡‡‡p<0.0001 (compared to Irbp-/-;Prss56-/- mice), one-way ANOVA. (TIF) [file pgen.1009458.s012.tif]

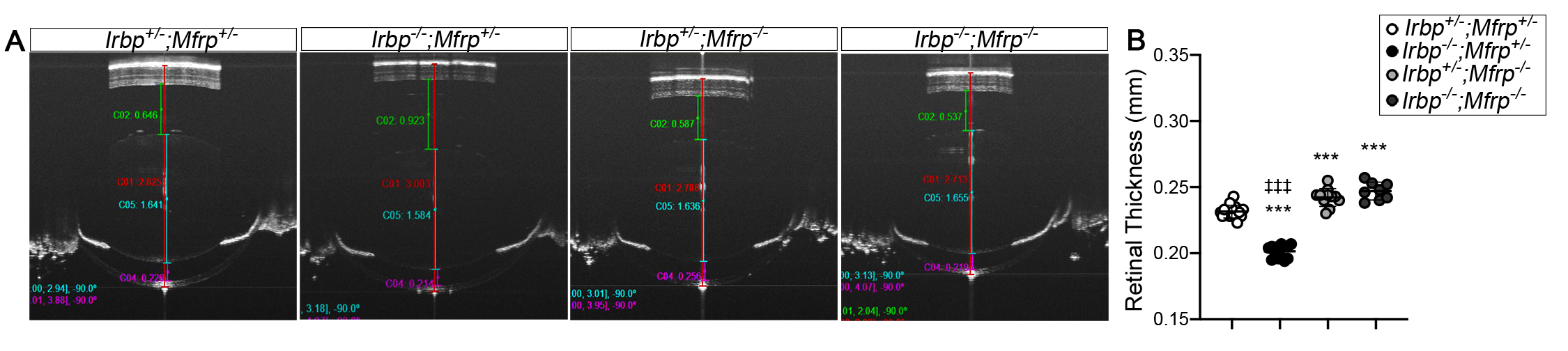

Supplement: S13 Fig — (A) Representative OCT images showing that ocular axial length (quantified in Fig 6C) and VCD (quantified in Fig 6D) are increased in Irbp mutant mice (Irbp-/-;Mfrp+/-) and reduced in Mfrp mutant and Irbp;Mfrp double mutant mice (Irbp+/-;Mfrp-/- and Irbp-/-;Mfrp-/-, respectively) compared to control Irbp+/-;Mfrp+/- mice at P18. (B) In contrast, and retinal thickness was reduced in Irbp mutant mice (Irbp-/-; Mfrp+/-) and increased in Mfrp mutant and Irbp;Mfrp double mutant mice (Irbp+/-;Mfrp-/- and Irbp-/-;Mfrp-/-, respectively) compared to control Irbp+/-; Mfrp+/- mice. Data are presented as mean ± SD, N≥8 eyes/group. ***p<0.0001 (compared to Irbp+/-; Mfrp+/- controls); ‡‡‡p<0.0001 (compared to Irbp-/-; Mfrp-/- mice), one-way ANOVA. (TIF) [file pgen.1009458.s013.tif]

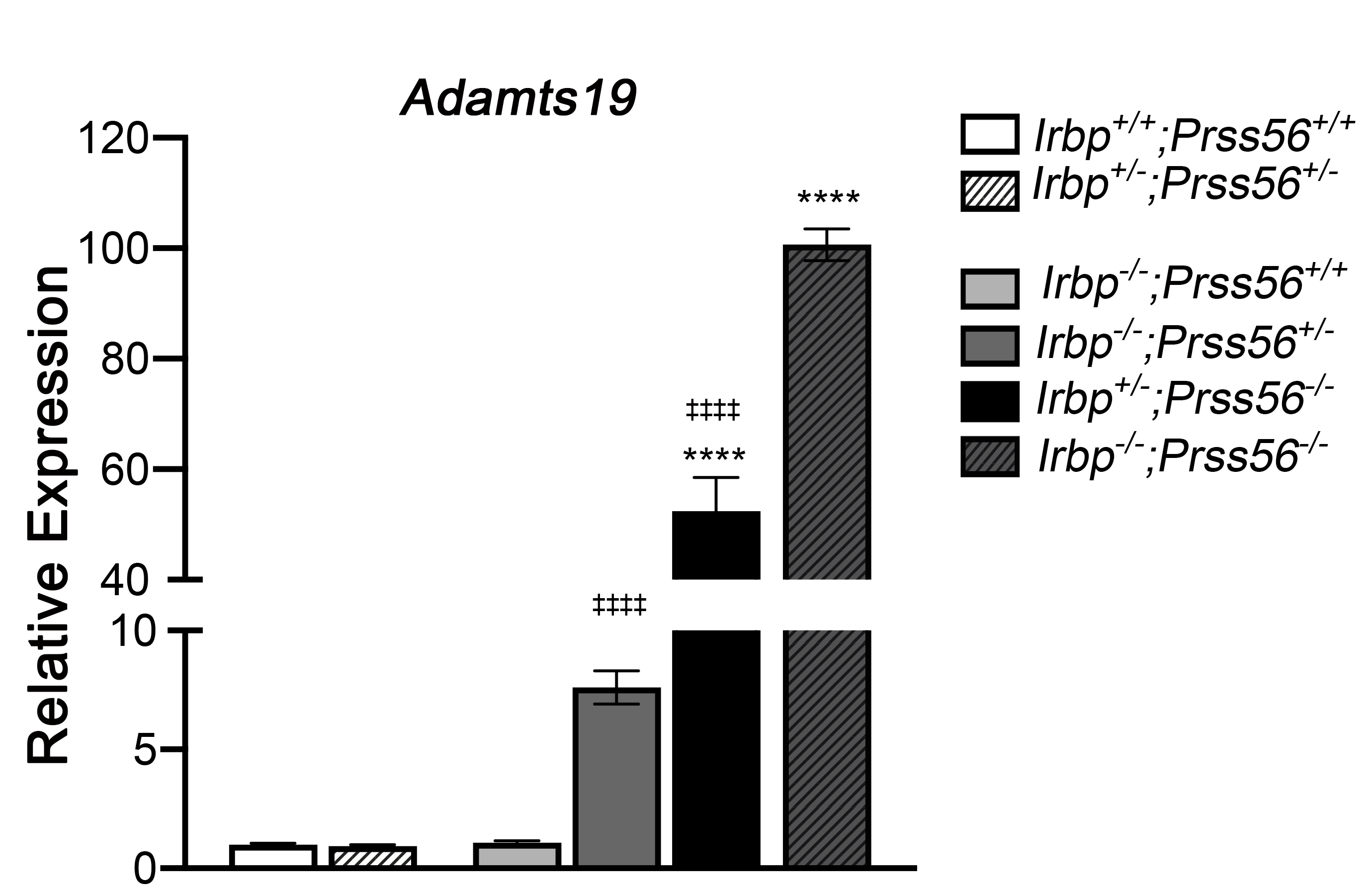

Supplement: S14 Fig — Graphs showing quantification of Adamts19 mRNA levels using qPCR analysis in the retina from P18 mice. Adamts19 expression was indistinguishable between wild-type (Irbp+/+;Prss56+/+), double heterozygous (Irbp+/-; Prss56+/-), and Irbp mutant mice carrying two wild-type alleles of Prss56 (Irbp-/-; Prss56+/+). In contrast, upregulation of retinal Adamts19 expression was observed in Irbp mutant mice that are heterozygous for Prss56 mutant allele (Irbp-/-;Prss56+/-). As expected, Adamts19 expression was significantly upregulated in Prss56 single mutant mice (Irbp+/-; Prss56-/-) compared to Irbp+/+;Prss56+/+ and Irbp+/-;Prss56+/- control mice. Interestingly, Adamts19 expression was further elevated in double mutant mice (Irbp-/-; Prss56-/-) compared to Prss56 single mutant mice (Irbp+/-; Prss56-/-). Data are presented as fold expression relative to control Irbp+/+;Prss56+/+ retina (mean ± SEM), N≥4 retinas /group. ***p<0.001; ****p<0.0001 (compared to control Irbp+/-; Prss56+/- mice); ‡‡‡‡p<0.0005 (compared to Irbp-/-; Prss56-/- mice), one-way ANOVA. (TIF) [file pgen.1009458.s014.tif]
